# Refining our understanding of depressive states and state transitions in response to cognitive behavioural therapy using latent Markov modelling

**DOI:** 10.1017/S0033291720002032

**Published:** 2022-01

**Authors:** Ana Catarino, Jonathan M. Fawcett, Michael P. Ewbank, Sarah Bateup, Ronan Cummins, Valentin Tablan, Andrew D. Blackwell

**Affiliations:** 1 Digital Futures Lab, Ieso Digital Health, The Jeffrey's Building, Cowley Road, Cambridge, CB4 0DS, UK; 2 Department of Psychology, Faculty of Science, Memorial University of Newfoundland, St John's, Canada

**Keywords:** Cognitive behavioural therapy, depression, depression sub-types, personalized medicine

## Abstract

**Background:**

It is increasingly recognized that existing diagnostic approaches do not capture the underlying heterogeneity and complexity of psychiatric disorders such as depression. This study uses a data-driven approach to define fluid depressive states and explore how patients transition between these states in response to cognitive behavioural therapy (CBT).

**Methods:**

Item-level Patient Health Questionnaire (PHQ-9) data were collected from 9891 patients with a diagnosis of depression, at each CBT treatment session. Latent Markov modelling was used on these data to define depressive states and explore transition probabilities between states. Clinical outcomes and patient demographics were compared between patients starting at different depressive states.

**Results:**

A model with seven depressive states emerged as the best compromise between optimal fit and interpretability. States loading preferentially on cognitive/affective *v.* somatic symptoms of depression were identified. Analysis of transition probabilities revealed that patients in cognitive/affective states do not typically transition towards somatic states and vice-versa. Post-hoc analyses also showed that patients who start in a somatic depressive state are less likely to engage with or improve with therapy. These patients are also more likely to be female, suffer from a comorbid long-term physical condition and be taking psychotropic medication.

**Conclusions:**

This study presents a novel approach for depression sub-typing, defining fluid depressive states and exploring transitions between states in response to CBT. Understanding how different symptom profiles respond to therapy will inform the development and delivery of stratified treatment protocols, improving clinical outcomes and cost-effectiveness of psychological therapies for patients with depression.

## Introduction

Decades of research have provided valuable insights into the nature of depression, with promising treatments emerging (Daly et al., 2019; The National Institute for Health & Care Excellence, 2009; Wijesinghe, 2014). However, whilst there is evidence that pharmacological approaches combined with psychotherapy comprise one of the most effective treatments for depressive disorders to date (Khan, Faucett, Lichtenberg, Kirsch, & Brown, 2012), only half of patients undergoing treatment recover (Holtzheimer & Nemeroff, 2006).

Existing diagnostic approaches are undoubtedly valuable in providing a unifying framework for patients with mental disorders and their clinicians. However, while useful for defining the primary presenting problem, current diagnostic systems may not be sufficient to explore the full range of human behaviour and to describe the rich underlying complexity of mental health disorders (Cuthbert & Insel, 2013). For example, by assuming a range of symptoms to be reflective of a singular underlying disease, diagnostic labels – considered in isolation – may mask considerable underlying heterogeneity within a given disorder. This is due to the fact that the classification of symptoms and the origin of diagnoses was built on expert consensus and the agglomeration of different phenotypes under the same diagnosis. Differences in symptom patterns within a condition and some degree of diagnostic overlap are therefore unavoidable. Although this is an issue for all mental health conditions, it is particularly problematic for depression. According to the DSM-5, amongst other diagnostic criteria, a diagnosis of major depressive disorder is suggested when a patient presents with five out of nine symptoms, one of which must be depressed mood or loss of interest or pleasure. This allows for a substantial degree of heterogeneity, as more than 100 combinations of symptom criteria can lead to the same unitary diagnosis of depression (Zimmerman, Ellison, Young, Chelminski, & Dalrymple, 2015). This assumption that depression is a homogenous entity may be an important reason behind treatment failures; i.e. the application of a ‘one size fits all’ approach to treatment, without regard for the latent phenotype expressed by a particular person. On this basis, research exploring different depression classes or subtypes within the broader definition of depression is widespread.

Clinicians have used various terms to distinguish between different manifestations of depression, including melancholic, atypical, anxious, psychotic, agitated and retarded depression (Goldberg, 2011; Insel, 2014; Lamers et al., 2016; O'Connor & Agius, 2015). The terminology varies widely based on various criteria, including symptom features (e.g. melancholic features) but also the time of onset, clinical history and comorbid symptoms of other mental health disorders (American Psychiatric Association, 2013). Not surprisingly this leads to a high degree of comorbidity, i.e. a patient can meet clinical criteria for more than one subtype or specifier, and the intensity of single symptoms is not usually considered, making it difficult for clinicians to navigate the wide range of different treatment options available and choose the most appropriate one for each case (Fried & Nesse, 2015; Goldberg, 2011; Linden & Rath, 2014; Musil et al., 2018). Despite this, studies have been conducted evaluating the effect of different treatments on each specifier or subtype. Unfortunately, results have been mixed and difficult to interpret (Arnow et al., 2015; Uher et al., 2011). Together with the fact that these definitions have not led to the development of subtype-specific treatment protocols, this throws into question the value of this classification system for determining the most appropriate treatment for a particular patient.

### Latent class and transition analyses of depression

In response to this, a number of data-driven approaches (i.e. approaches where theoretical constructs are not enforced upon the statistical model a priori) have arisen recently using techniques such as latent class analysis (LCA) or latent profile analysis (LPA) to identify depression subtypes on the basis of observable responses (e.g. taken from a diagnostic questionnaire, without recourse to subjective judgments) (Putnam et al., 2015; Ulbricht, Chrysanthopoulou, Levin, & Lapane, 2018a; Ulbricht, Rothschild, & Lapane, 2015). Within health research, these approaches have proven useful in clustering patients across a range of multidimensional symptoms and disorders (for a summary of LCA in health research, see Kongsted and Nielsen, 2017).

However, LCA has been relatively less consistent in drawing strong qualitative distinctions amongst depression subtypes. Early studies adopting this technique have tended to identify subtypes that differ on the basis of severity rather than qualitative response profiles (for a recent review, see Ulbricht et al., 2018a). Whereas more recent work has continued to support severity as a major indicator, the distinction between cognitive-affective and psychosomatic symptoms has increasingly been identified as playing an important role (Barton, 2017; Carragher, Adamson, Bunting, & McCann, 2009; Chen, Eaton, Gallo, & Nestadt, 2000; Lee et al., 2012; Lee, Stroo, Fuemmeler, Malhotra, & Østbye, 2014). Cross-sectional approaches such as factor analysis, which reveal how symptoms cluster together for a given metric such as the Patient Health Questionnaire (PHQ-9), provide further support to the idea that cognitive-affective and somatic symptoms may reflect unique latent variables within depression (Chilcot et al., 2013; Doi, Ito, Takebayashi, Muramatsu, & Horikoshi, 2018; Krause, Reed, & McArdle, 2010). This distinction may be particularly relevant when evaluating the effectiveness of cognitive behavioural therapy (CBT) protocols targeting cognitive *v.* somatic features of depression.

Despite advancing our knowledge of depression, the use of LCA has been limited to rigid clustering of patients into static classes, providing no indication of how different depression subtypes evolve over time or in response to treatment. A better understanding of how patients in different classes respond to treatment is essential in promoting the delivery of personalized treatment protocols, with the aim of improving clinical outcomes for patients. Latent transition analysis (LTA) is an extension of LCA which uses longitudinal data to explore transitions between classes over time. However, this technique has not been applied broadly within the area of depression research: as with LCA, most LTA studies report a classification of depressive subtypes based on severity, with transition analyses focusing on whether patients transitioned to symptom resolution states or show symptom stability over time (Ni, Tein, Zhang, Yang, & Wu, 2017; Tay, Jayasuriya, Jayasuriya, & Silove, 2017; Tisminetzky, Bray, Miozzo, Aupont, & McLaughlin, 2011). Although some studies also report classification of depression subtypes according to clinical features (e.g. psychomotor disturbances, changes in appetite, insomnia), small sample sizes and the overall confounding effect of severity mean that consensus across studies and patient cohorts remains poor (Ulbricht et al., 2015; Ulbricht, Dumenci, Rothschild, & Lapane, 2016, 2018b). This results in a wide range of findings with limited interpretability and applicability to improving clinical care in the future (see Li et al., 2014 for a summary table of 16 studies using latent class analysis to subtype depression).

The aim of this study was to identify depressive states in a large-scale patient population and to explore how different symptom profiles respond to psychotherapy while controlling for overall severity. This study represents the first application of LTA to isolate latent depressive states and characterize transitions amongst them, within a large-scale patient population receiving a course of internet-enabled Cognitive Behavioural Therapy (IECBT). With a strong evidence base, CBT is the most common psychological therapy used to treat depression in the USA and the UK. In IECBT, a patient communicates with a qualified CBT therapist using a real-time text-based message system. IECBT has been shown to be clinically effective for the treatment of depression (Kessler et al., 2009) and is currently deployed within the English National Health Service. By understanding how different symptom profiles respond to therapy, it may be possible to develop and deliver personalized treatment protocols with the aim of improving treatment outcomes for patients with depression.

## Methods

Data were obtained from patients receiving IECBT, delivered using a commercial package provided by Ieso Digital Health (https://www.iesohealth.com/), following internationally recognized standards for information security (ISO 27001; https://www.iesohealth.com/en-gb/legal/iso-certificates). Patients self-referred or were referred by a primary healthcare worker directly to the service. Upon registration, patients were assigned to a qualified CBT therapist accredited by the British Association for Behavioural & Cognitive Psychotherapies (BABCP). Initial assessments and NICE approved disorder-specific CBT treatment protocols (The National Institute for Health & Care Excellence, 2009), based on Roth and Pilling's CBT competences framework (Roth & Pilling, 2008), were delivered during scheduled sessions in an online therapy room, via one-to-one real-time written conversation.

The Improving Access to Psychological Therapies (IAPT) program, under which Ieso Digital Health operates, is a large-scale national initiative aimed at increasing access to evidence-based psychological therapy for common mental health disorders within the English NHS (Clark, 2011). The information captured through IAPT's minimum dataset, including IECBT, is intended to support monitoring of implementation and effectiveness of national policy/legislation, policy development, performance analysis and benchmarking, national analysis and statistics and national audit of IAPT services. At registration, patients agree to the services' terms and conditions, including the use of anonymized data for audit purposes and to support research, including academic publications or conference presentations.

### Clinical outcomes

Clinical outcomes were measured in terms of *IAPT-engagement*, *reliable improvement*, *per cent improvement* and *deterioration*, and were included as binary measures (i.e. 0 or 1). Following IAPT guidelines a patient was classed as *engaged* if they attended two or more treatment sessions. This is the minimum dose of therapy a patient must receive such that pre- and post-treatment scores are collected and clinical change can be estimated (Gyani, Shafran, Layard, & Clark, 2013). Clinically *reliable improvement*, *per cent improvement* and *deterioration* are calculated based on two severity measures completed by the patient at initial assessment and before every therapy session: PHQ-9 (Kroenke, Spitzer, & Williams, 2001) and GAD-7 (Spitzer, Kroenke, Williams, & Löwe, 2006), corresponding to depressive and anxiety symptoms, respectively.

Patients with two or more therapy sessions who show a significant reduction in at least one of the outcome measures from assessment to the last treatment session (i.e. decrease of six points or more in the PHQ-9 and/or four points or more in the GAD-7), while not showing a significant increase in the other outcome measure (i.e. an increase of six points or more in the PHQ-9 or four points or more in the GAD-7), were classed as showing *reliable improvement*. Patients showing a significant increase in at least one of the outcome measures from assessment to last treatment session (i.e. increase of six points or more in the PHQ-9 or four points or more in the GAD-7), were classed as showing *deterioration*.

Similar to IAPT convention, we classed a patient as achieving *per cent improvement* if they showed a 25% decrease in scores in one or both scales, without showing symptom worsening in either scale (i.e. 25% increase in scores in either scale). IAPT's improvement metric is, by definition, biased by initial symptom severity, i.e. more severe patients are more likely to improve due to their initial higher scores. *Per cent improvement* has the advantage of reducing this bias while retaining similar properties to the IAPT-improvement metric, in the sense that it is a binary measure that reflects the change in scores from start to end of treatment (Hiller, Schindler, & Lambert, 2012). While *per cent improvement* may reduce bias for more symptomatic patients, it naturally introduces a small bias for less symptomatic patients. Nevertheless, considered alongside each other, these two metrics provide a more accurate representation of patients' response to treatment, relative to either metric considered in isolation.

### Sample size

More than 48 000 patients were discharged from the IECBT service between June 2012 and January 2019. Of these, 10 795 received a diagnosis of depression, recurrent depression disorder or dysthymia from a qualified clinician, and met inclusion criteria for the service (over 18 years old, registered with a general practitioner in the geographical region where the service is commissioned, not at significant risk of self-harm and no presence of an axis II disorder). A total of 9891 patients with at least one PHQ-9 score, collected at initial assessment, but no more than 10 scores were included in the analysis. The latter criterion was instated to keep the computational demands of our modelling approach manageable; importantly, few patients had more than 10 scores (8%). Of the patients included in the analysis, 6958 (70%) attended two or more therapy sessions (IAPT engaged) and were therefore included in analyses on clinical improvement outcomes. Patients with only one PHQ-9 score were included to inform the model at timepoint 1 (i.e. when patients present to the service), which in turn informs our understanding of the model and transitions for subsequent timepoints.

### Modelling depressive states

**Fig. 1.**
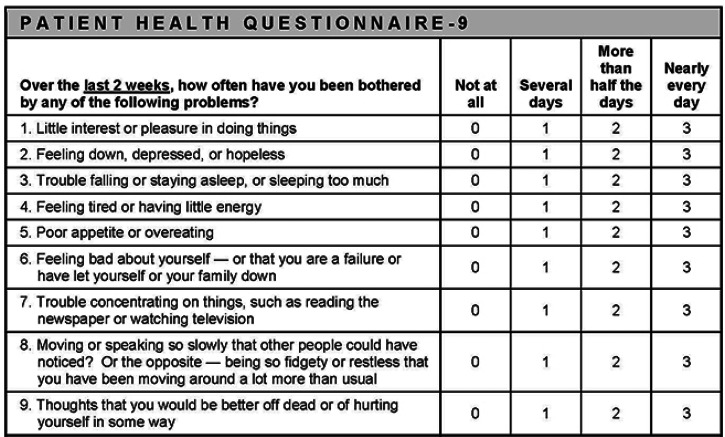
Patient health questionnaire (PHQ-9).

Even though PHQ-9 and GAD-7 are used in combination to assess clinical improvement within the IAPT program, variations in clinical presentation for depressed patients are more likely to be captured by the PHQ-9 questionnaire (Fig. 1).

**Fig. 2.**
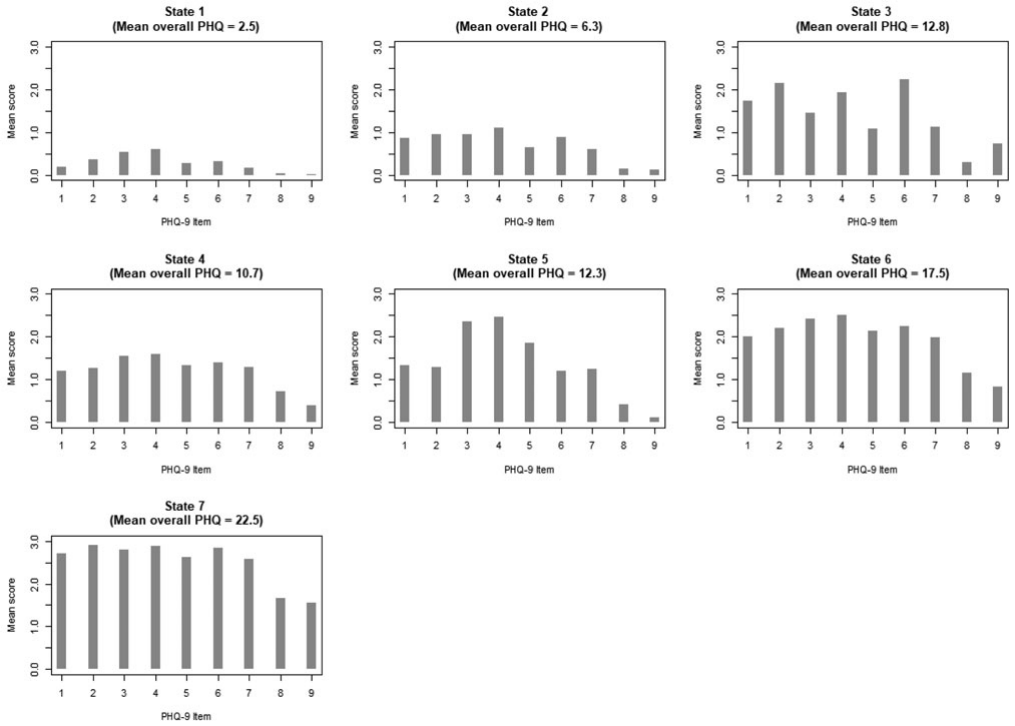
Graphical summary of state symptom profiles for the 7-state model. States 1 and 2 represent states of minimal to mild overall severity; State 3 shows peak symptom intensity around feelings of depression, tiredness and low self-esteem (cognitive/affective state); State 5 shows peak symptom intensity around difficulties sleeping, feelings of tiredness, and changes in appetite (somatic state); State 4 shows a relatively even spread in symptom intensity across items (hybrid state); States 6 and 7 represent moderately severe and severe states, respectively.

Item-level PHQ-9 scores, collected at registration and before each therapy session for all patients, were used as input to a Hidden Markov Model (HMM) implemented using the *LMest* (Bartolucci, Pandolfi, & Pennoni, 2017) package in *R* v.3.5.0 (R Core Team, 2018) to estimate latent states and transition probabilities between states. In the reported models, we assumed heterogeneity of transition probabilities across time – allowing for the possibility that specific state-to-state transitions become more likely (or unlikely) throughout the treatment process. Models were fitted assuming a number of states ranging from 1 through 16, with the final number of states selected based on the corresponding Bayesian Information Criterion (BIC). This approach revealed empirical support for 14 states. However, an inspection of the resulting profiles revealed several states as representing minor empirical differences (e.g. gradations of severity) rather than meaningful, qualitative distinctions. We believe these gradations in severity are of less interest, and for that reason, we chose to interpret a model with seven states as a compromise between optimal model fit and interpretability (Fig. 2). We have run the 7-state model on three independent folds of our data, each replicating similar states and transition probabilities (see online Supplementary Figs S4 and S5). This supports the hypothesis that the 7-state latent structure presented is not spurious. For transparency, the full 14-states model and transition probabilities supported by the data are also detailed in Supplementary Materials (see online Supplementary Figs S1, S2 and S3 and Supplementary Table S2). Following the model fit, the depressive state at each time point was estimated for all patients using global decoding. Decoded data were used to explore state transitions over time and in post-hoc analyses to evaluate differences in patient demographics and clinical outcomes, based on starting state.

### Statistical analyses

The PHQ-9 has been demonstrated to be comprised of two factors, one loading on somatic symptoms (e.g. difficulties sleeping, tiredness, changes in appetite), and one loading on cognitive/affective symptoms (e.g. feeling down and depressed, low self-esteem) (Chilcot et al., 2013; Doi et al., 2018; Krause et al., 2010). Post-hoc analyses investigating differences in clinical outcomes and demographics, therefore, focused on depressive states loading more markedly on the cognitive/affective factor (State 3) and somatic factor (State 5). We first performed statistical analyses to investigate differences in outcomes and demographics between patients presenting to treatment in State 3 and State 5.

Pearson's χ^2^ tests were performed to compare rates of IAPT-engagement for patients entering treatment at State 3 or State 5 (*n* = 2959 patients: State 3: 1685; State 5: 1274), and to compare rates of reliable improvement, deterioration and per cent improvement for engaged patients (*n* = 2092: State 3: 1234; State 5: 858).

Logistic regression was then performed to investigate which patient demographics were predictive of state. Starting state was included as a binary outcome measure (State 3 = 1, State 5 = 0). Predictor variables were patient age, gender, sexual orientation, ethnicity, religion, whether the patient was in the perinatal period, whether the patient suffered from a long-term physical condition, whether the patient was taking psychotropic medication at the start of treatment, whether the patient was in active military service and whether the patient had a disability. PHQ-9 and GAD-7 scores at assessment were also included as continuous variables to account for any baseline differences in symptom severity. Continuous predictor variables were scaled and centred to the mean. Statistical significance was defined as *p* < 0.05 two-tailed, uncorrected. Multicollinearity analyses revealed that variance inflation factors were smaller than two for all predictor variables, confirming that the regression model was not affected by the presence of multicollinearity. All analyses were performed in R.

## Results

Our chosen HMM revealed seven depressive states with varied symptom profiles and differing overall severity levels (Fig. 2) (see Methods). States 1 and 2 represent states of minimal to mild overall severity, with low intensity across all items. States 3, 4 and 5 represent states of moderate severity, but differing symptom profiles. While State 4 shows a relatively even spread in symptom intensity across items, State 3 shows peak intensity for items centred around feelings of depression, tiredness and low self-esteem (cognitive/affective state). State 5 shows peak intensity for items centred around difficulties sleeping, feelings of tiredness and changes in appetite (somatic state). States 6 and 7 represent moderately severe and severe states respectively, both showing evenly high intensity across items.

**Fig. 3.**
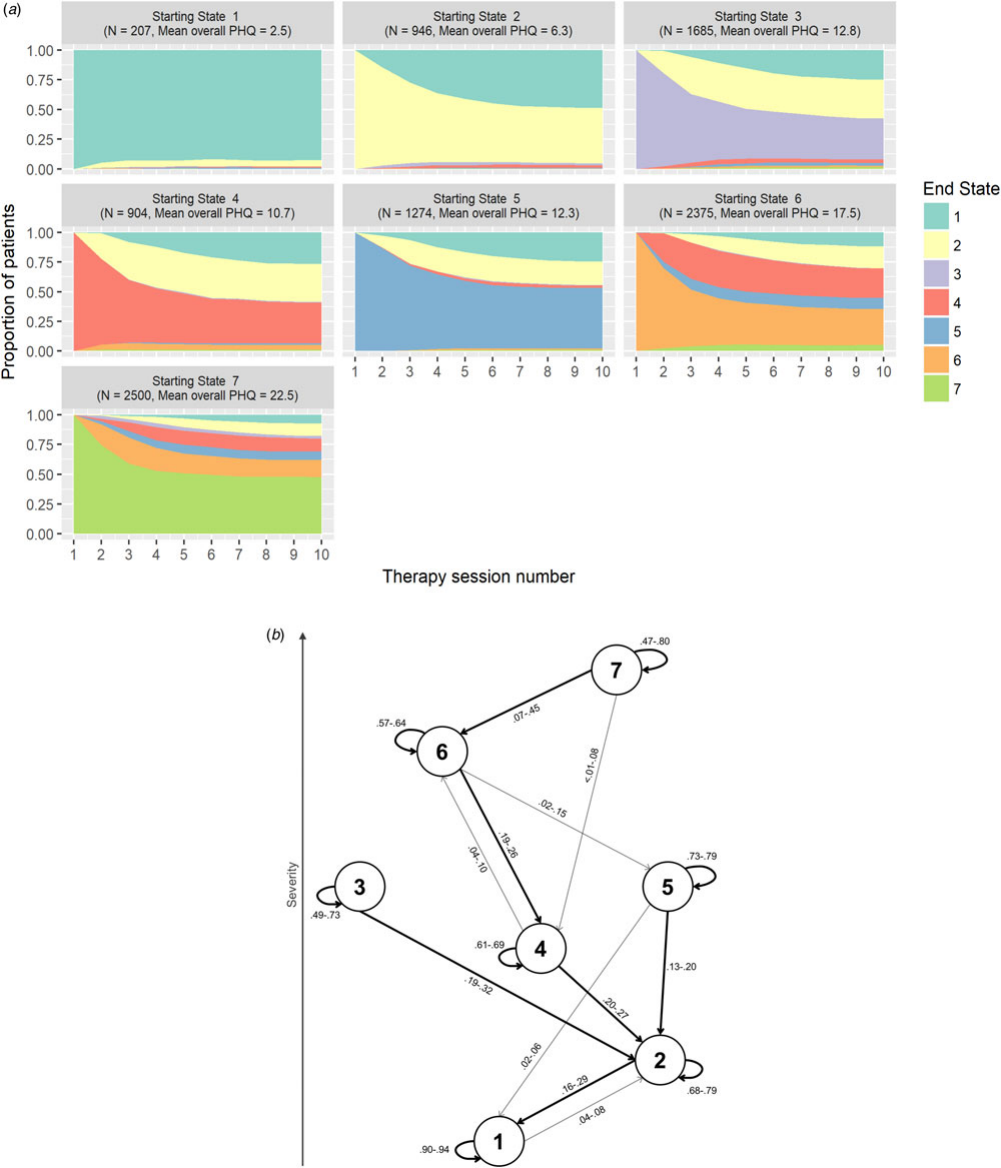
(*a*) Stacked area plots showing transitions between states over time for each starting state; patients leaving treatment were considered to remain at whatever state they last exhibited. (*b*) Transition probability graph showing the range of transition probabilities across time for each depressive state; transition probabilities below 0.05 for more than half of the time points are omitted; thicker arrows represent the most likely transitions between two given states. A full transition probability matrix is available in online Supplementary Materials.

The model used in the current study assumes heterogeneity of transition probabilities across time. To illustrate state progression over time, we estimated the probable state of each patient at each time point, plotted as a function of their initial state (Fig. 3*a*). A transition probability graph, showing a range of transition probabilities across time, is also shown in Fig. 3*b*. The full 3-dimensional transition probability matrix (‘starting state’ by ‘end state’ by ‘time’) can be found in Supplementary Materials (online Supplementary Table S3). Overall, most patients tended to either remain in their initial state, or transition to a state of lower overall severity, as would be expected in response to a therapeutic intervention. It is interesting to note however that these transitions are not homogeneous across starting states. For example, despite its lower overall symptom severity, State 4 is bypassed by patients starting in cognitive/affective State 3 and somatic State 5, as they progress to recovery. On the other hand, patients starting in more severe States 6 and 7 do seem to transition to State 4 as they progress through therapy but are less likely to transition to somatic State 5, and almost never transition to cognitive/affective State 3. A similar pattern is observed for patients experiencing worsening of their symptoms, where a small proportion of patients starting in State 4 deteriorate towards State 6, but not to somatic State 5 or cognitive/affective State 3. It is also interesting to observe differences across starting states in terms of patients' likelihood to remain in their starting state. For example, while only around a quarter of patients starting at States 3, 4 and 6 remain at their initial state after a course of therapy, approximately half of patients starting in States 5 and 7 do so.

### Cognitive/affective and somatic depression: association with outcomes and demographics

A chi-square test comparing the rate of IAPT-engagement in cognitive/affective (State 3) and somatic (State 5) states revealed significant differences, with higher engagement rates found for patients entering treatment in a cognitive/affective state [χ^2^(12 959) = 11.86, *p* < 0.001]. Additional chi-square tests revealed that both the rate of reliable improvement and per cent improvement were also significantly higher for patients starting treatment in cognitive/affective relative to somatic state [Improvement: χ^2^(12 092) = 11.64, *p* < 0.001; Per cent improvement: χ^2^(12 092) = 4.17, *p* = 0.041; Table 1]. No significant difference in deterioration rates was observed between patients starting treatment in cognitive/affective and somatic states (χ^2^(12 092) = 1.65, n.s.).

**Table 1.** Engagement and clinical outcomes for each starting state

Start State	N	IAPT-engagement (%)	Mean number sessions (s.d.)	Start PHQ-9	Improvement (%)	Per cent improvement (%)	Deterioration (%)
1	207	68.6	4.4 (2.6)	3.0 (1.8)	22.5	66.9	9.9
2	946	72.6	4.7 (2.5)	6.6 (1.5)	49.3	82.5	6.8
3	1685	73.2	5.2 (2.6)	13.2 (3.0)	81.6	90.8	7.9
4	904	76.5	5.2 (2.5)	10.9 (1.7)	65.6	81.5	10.1
5	1274	67.3	4.6 (2.7)	12.7 (2.8)	74.4	86.1	10.7
6	2375	73.4	5.2 (2.7)	17.7 (2.2)	82.3	86.0	11.0
7	2500	64.0	4.7 (2.8)	22.4 (2.5)	73.5	71.5	7.8

A logistic regression investigating the relationship between starting state and patient demographics revealed a significant relationship between the state and patient gender, the presence of a long-term medical condition and medication status (Table 2). Patients entering treatment in a somatic state were significantly more likely to present with lower overall symptom severity (PHQ-9 and GAD-7) and more likely to be female, be prescribed and taking medication, and have a long-term medical condition.

**Table 2.** Results of logistic regression analysis investigating the relationship between patient demographics and starting state [cognitive/affective (State 3) or somatic (State 5)]

Predictor variable	Mean/prevalence State 3 State 5		*b*	s.e.	Wald's statistic, *z*^2^	*p*
Start PHQ-9 score, mean	13.2	12.7	0.11	0.05	6.16	0.013 *
Start GAD-7 score, mean	10.7	9.9	0.19	0.05	18.04	<0.001***
Patient age, years: mean	35.8	36.7	−0.03	0.05	0.31	0.578
Gender, %						
Male	35.9	23.2	0.69	0.10	51.11	<0.001***
Unknown/not stated	0.2	0.4	−0.46	0.68	0.46	0.496
Sexual orientation, %						
Homosexual/Bisexual	8.4	7.6	0.12	0.16	0.57	0.451
Unknown/not stated	11.7	11.0	−0.16	0.19	0.74	0.390
Long term condition, %						
Yes	24.7	29.3	−0.22	010	4.49	0.034*
Unknown/not stated	25.3	24.0	−0.03	0.11	0.09	0.758
Psychotropic medication, %						
Prescribed not taking	7.7	6.5	0.05	0.17	0.09	0.768
Prescribed taking	35.4	42.4	−0.29	0.09	10.50	0.001**
Unknown/not stated	1.5	1.9	0.01	0.35	0.001	0.972
Ethnicity, %						
White	84.9	84.9	0.21	0.16	1.74	0.187
Unknown/not stated	8.0	6.7	0.08	0.30	0.07	0.795
Religion, %						
None	41.4	36.1	0.11	0.10	1.21	0.271
Other	5.4	7.6	−0.32	0.18	3.10	0.078
Unknown/not stated	22.4	21.9	−0.02	0.13	0.02	0.900
Military, %						
Yes	1.3	1.7	−0.46	0.35	1.77	0.184
Unknown/not stated	9.6	7.5	0.43	0.24	3.30	0.069
Perinatal, %						
Yes	5.1	7.0	−0.30	0.18	2.89	0.089
Unknown/not stated	0.7	0.5	0.15	0.53	0.09	0.771
Disability, %						
Yes	13.3	16.2	−0.19	0.12	2.38	0.123

A positive relationship indicates that a variable is significantly more likely to occur in patients starting in the cognitive/affective state (State 3). Gender ‘Female’, sexual orientation ‘Heterosexual’, long term condition ‘No’, psychotropic medication ‘Not Prescribed’, ethnicity ‘Non-White’, religion ‘Christian’, military ‘No’, perinatal ‘No’ and disabled ‘No’ were reference classes for the categorical variables ***p < 0.001, **p < 0.01, *p < 0.05.

## Discussion

To our knowledge, this is the first paper to isolate depression states and characterize transitions between those states using LTA in a large sample of patients receiving CBT. This study represents a novel advancement, by providing an investigation of different depression states in a real-world clinical setting, and their response to a course of CBT.

### Main findings

HMM analysis of item-level PHQ-9 data revealed seven depressive states varying in symptom profile and overall severity. While States 1, 2 and 7 represent minimal, mild and severe states respectively, with symptom severity either at the floor or at the ceiling, moderate to moderate-severe states (State 3 to 6) demonstrate interesting variations in item-level severity (Fig. 2). Cross-sectional approaches such as factor analyses explore how certain symptoms cluster together for a given metric. Although these types of analyses provide no information on how different symptom clusters are associated with or interact with each other, they provide an interesting context on which to interpret the current findings. The depressive states described here can be considered in the context of research demonstrating a two-factor structure for the PHQ-9 scale, separating somatic and cognitive/affective symptoms (Chilcot et al., 2013; Doi et al., 2018; Krause et al., 2010). For example, the most severe symptoms for State 3 appear to load on the cognitive/affective factor of the PHQ-9, while the most severe symptoms for State 5 load on the somatic factor of the scale. In States 4 and 6, patients' symptoms seem to load equally on both cognitive/affective and somatic factors (hybrid states). This distinction between cognitive/affective and somatic depressive symptoms is further supported by previous research exploring depression subtyping using latent classification analysis (Carragher et al., 2009). Interestingly, to our knowledge, our study is the first to demonstrate a similar latent structure using LTA, and therefore the first to characterize transitions between these subtypes.

This work also shows how patients starting in each of these states transition between states over a course of therapy. It is interesting to note that state transitions seem to stabilize at around treatment session 6. This is likely a reflection of the mean treatment duration across all starting states (i.e. approximately five treatment sessions), but also that in CBT the greatest clinical benefit is likely to be achieved in the first half of treatment (Ilardi & Craighead, 1994; Tang & DeRubeis, 2006). It is also interesting to observe that patients in the cognitive/affective and somatic states (State 3 and 5 respectively) do not transition to hybrid State 4, despite this being lower in overall severity (Fig. 3*a* and 3*b*). Similarly, about a quarter of patients starting in hybrid State 6 transition to hybrid State 4, with a small probability of transition to less severe States 3 or 5. A similar pattern is observed for symptom deterioration, where patients starting in hybrid State 4 deteriorate with low probability to hybrid State 6, but not to cognitive/affective and somatic States 3 and 5. Patients in cognitive/affective State 3 and somatic State 5 also do not seem to deteriorate to hybrid States 6 or 7. Differential loading on the cognitive/affective *v.* somatic factors of the PHQ-9 metric, together with differences in transition probabilities across states, provide initial evidence for the existence of different depression subtypes.

We further explored this hypothesis by evaluating differences in clinical outcomes and patient demographics across cognitive/affective and somatic states. Despite similar overall severity, patients starting treatment in cognitive/affective and somatic states (States 3 and 5) show significant differences in outcomes, with somatic patients less likely to engage with treatment, improve or show per cent improvement. Related to this, we note that patients starting in hybrid State 6 show a small probability of transitioning to somatic State 5, but not to cognitive/affective State 3. Together, this may suggest that IECBT (or CBT in general) may be more effective at targeting cognitive/affective symptoms, with somatic symptoms appearing to be more resistant to treatment – the literature suggests that treatments targeting maladaptive cognitions are sufficient to improve symptoms for some patients, whereas for others this approach is significantly less effective (Hayes, 2016; Kazdin, 2007; Lorenzo-Luaces, German, & DeRubeis, 2015).

A regression analysis on patient demographics also revealed that patients who start in a somatic state are more likely to be female, suffer from long-term physical comorbidity, and be taking psychotropic medication (Table 2). These findings suggest demographic and clinical differences between the two states that go beyond mental health presentation, although the nature of the causal relationship between demographic variables and depressive state remains unclear. For example, it can be hypothesized that female patients with a long-term physical condition share a physiological substrate that makes them more likely to develop depression with somatic features. On the other hand, the prevalence of somatic symptoms in patients with long-term physical comorbidity is also expected to some degree, as some of these comorbidities can be associated with physical symptoms, such as persistent lack of energy and tiredness. Equally, patients who are prescribed antidepressants and anxiolytics are more likely to suffer from somatic symptoms (which can include fatigue, insomnia and changes in appetite) as a consequence of medication side-effects. It can be noted that the regression analysis also revealed that patients who start in a cognitive/affective state show higher overall symptom severity for both the PHQ-9 and GAD-7 scales. However, this association is unlikely to be clinically meaningful (as supported by a difference of less than 1 in the group average for both scales), with its significance being inflated by the large size of the sample.

Overall, this study provides important preliminary evidence for the existence of different depression subtypes, characterized by depressive states with different symptom profiles and different transition probabilities between states. Differences in clinical outcomes and demographics between patients in cognitive/affective *v.* somatic depressive states further support this hypothesis.

Data-driven approaches such as the one used in this paper do not fully address the weaknesses of existing classification systems, such as symptom and diagnostic overlap. Indeed, the interpretation of the results of these data-driven approaches is still informed by existing theories on diagnostic classification. Nevertheless, we believe the current model presents remarkable clinical potential – possibly implemented as part of a digital triage tool – which would allow clinicians to identify patients in depressive states which are typically less responsive to therapy. This would then enable the development and deployment of pharmacological and psychotherapeutic interventions in a stratified manner, aimed at increasing engagement and addressing core symptoms of a patient's condition, potentially improving their likelihood of responding to treatment.

The development of stratified treatment interventions also merits further research investigating the effect of therapeutic features (e.g. therapist effects, therapeutic content) on transition probabilities between depressive states. Better targeted interventions would have the dual advantage of improving clinical outcomes, as well as improving the cost-effectiveness of psychological therapies. Finally, future research should also investigate the generalizability of these models to clinical populations receiving other types of therapy (e.g. face-to-face CBT, psychodynamic therapy), or patients presenting with depressive symptoms as a secondary problem (e.g. primary presenting mental health condition with comorbid depressive features).

In this light, the present study not only deepens our knowledge of depression as a mental health disorder but by exploring the dynamic response to therapy in different depression subtypes also raises interesting possibilities for future research and the development of stratified treatment interventions aimed at improving clinical outcomes in patients with depression.

## Supplementary material

For supplementary material accompanying this paper visit https://doi.org/10.1017/S0033291720002032. click here to view supplementary material
