# Supplementary material for: Refining our understanding of depressive states and state transitions in response to cognitive behavioural therapy using latent Markov modelling
Source: Psychol Med. 2020 Jun 29;52(2):332–41. doi: 10.1017/S0033291720002032 (PMC8842194; doi:10.1017/S0033291720002032)
Supplement: Supplementary file 1 [file S0033291720002032sup001.docx]

| **N states** | **AIC** | **AIC difference** | **BIC** | **BIC difference** |
| --- | --- | --- | --- | --- |
| 1 | 1594979.5 | NA | 1595172.6 | NA |
| 2 | 840772.9 | -754206.6 | 841280.6 | -753892.0 |
| 3 | 793082.4 | -47690.5 | 794019.0 | -47261.5 |
| 4 | 774750.7 | -18331.7 | 776230.7 | -17788.3 |
| 5 | 767260.5 | -7490.2 | 769398.3 | -6832.4 |
| 6 | 758044.9 | -9215.6 | 760954.9 | -8443.5 |
| 7 | 752056.3 | -5988.6 | 755852.8 | -5102.0 |
| 8 | 747646.5 | -4409.8 | 752444.0 | -3408.8 |
| 9 | 743812.3 | -3834.2 | 749725.2 | -2718.8 |
| 10 | 739846.9 | -3965.4 | 746989.6 | -2735.5 |
| 11 | 736814.0 | -3032.9 | 745300.9 | -1688.7 |
| 12 | 734390.3 | -2423.7 | 744335.8 | -965.1 |
| 13 | 731995.4 | -2394.9 | 743513.8 | -822.0 |
| **14** | **729862.7** | **-2132.7** | **743068.5** | **-445.3** |
| 15 | 728265.2 | -1597.5 | 743272.7 | 204.2 |
| 16 | 727340.1 | -925.1 | 744263.7 | 991.0 |

**Supplementary materials**

**Supplementary Table 1 –** Full model details showing Akaike information criterion (AIC) and Bayesian information criterion (BIC) for N states. Results show a model with 14 states minimises BIC. A graphical summary of each state for the best fitting 14-state model is shown in supplementary Figure 1.


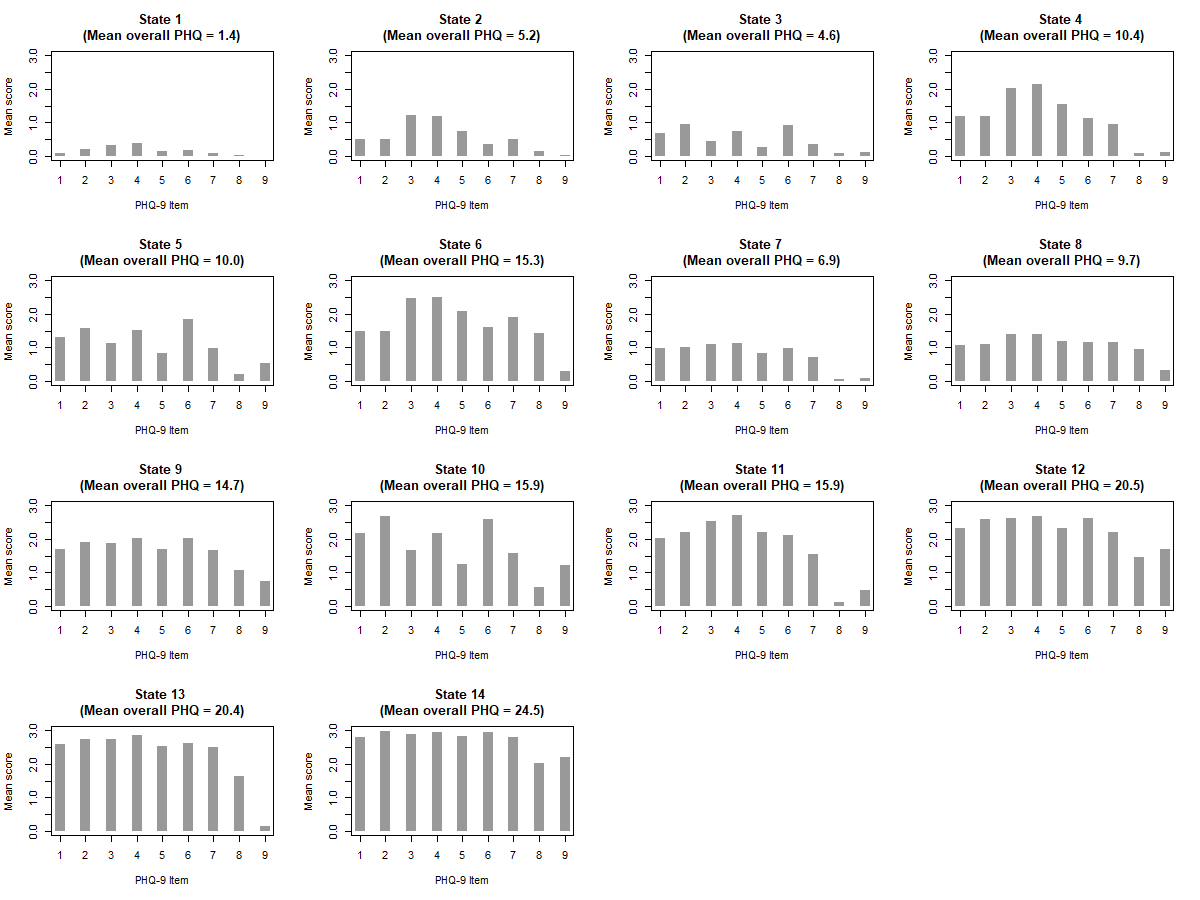
**Supplementary Figure 1 –** Graphical summary of state symptom profiles for the best fitting 14-state model. Gradations in severity can be observed – State 1 represents a state of minimal severity (corresponding to State 1 in the 7-state model); States 3, 5 and 10 show the same cognitive/affective symptom profile with different degrees of overall severity (corresponding to single State 3 in the 7-state model); States 2, 4, 6 and 11 show the same somatic symptom profile with different degrees of overall severity (corresponding to single State 5 in the 7-state model); States 7, 8 and 9 show relatively even severity across symptoms in the mild range (corresponding to State 4 in the 7-state model); States 12, 13 and 14 show even severity across symptoms in the moderate/severe range (corresponding to States 6 and 7 in the 7-state model). Aside from gradations in severity, minor symptomatic differences are also observed – e.g. States 12 and 14 differ from State 13 in suicidal ideation (PHQ-9 item 9); States 6 and 11 differ from each other in psychomotor retardation/agitation (PHQ-9 item 8).


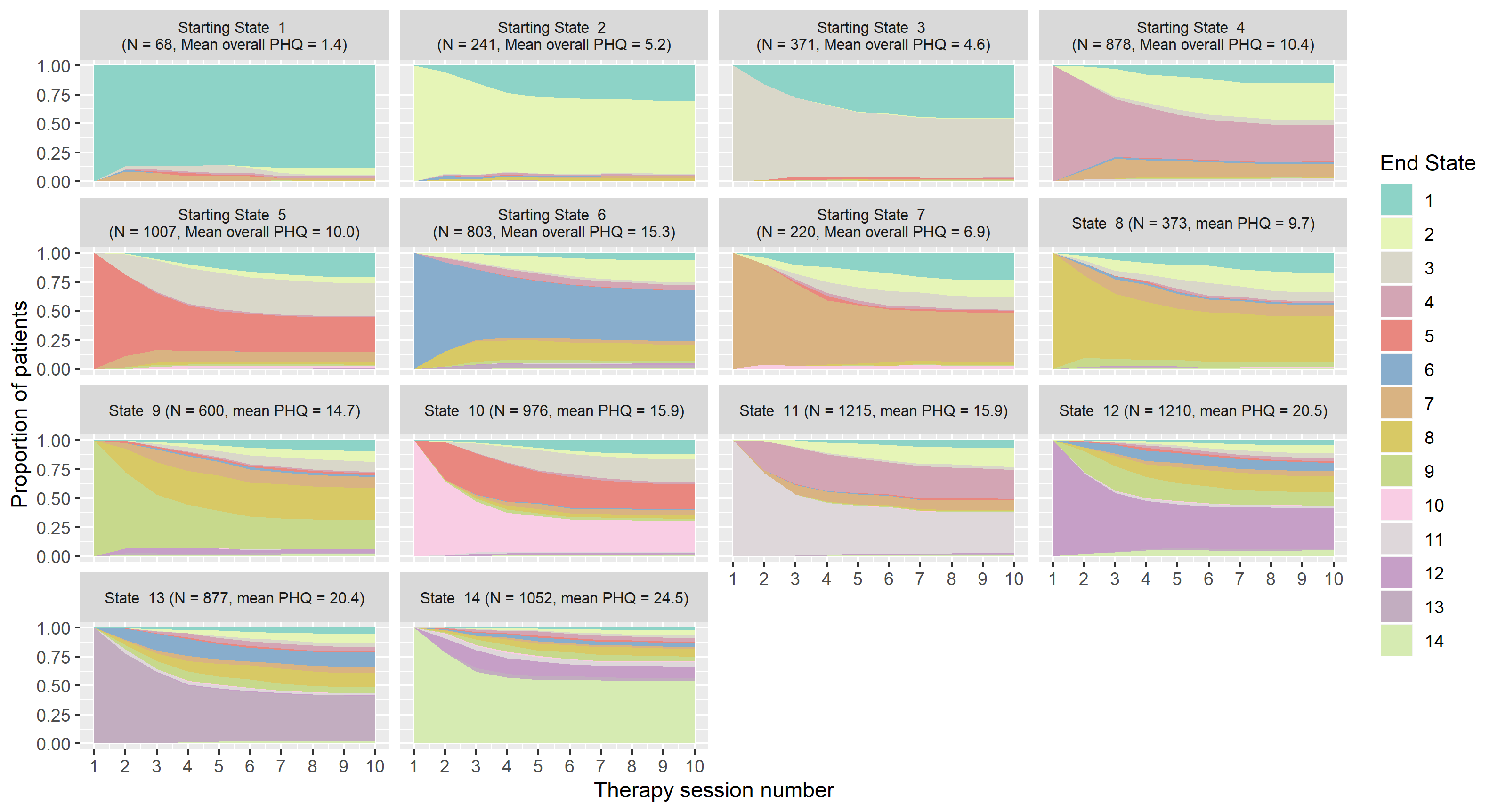


**Supplementary Figure 2 –** Stacked area plots showing transitions between states over time for each starting state; patients leaving treatment were considered to remain at whatever state they last exhibited.


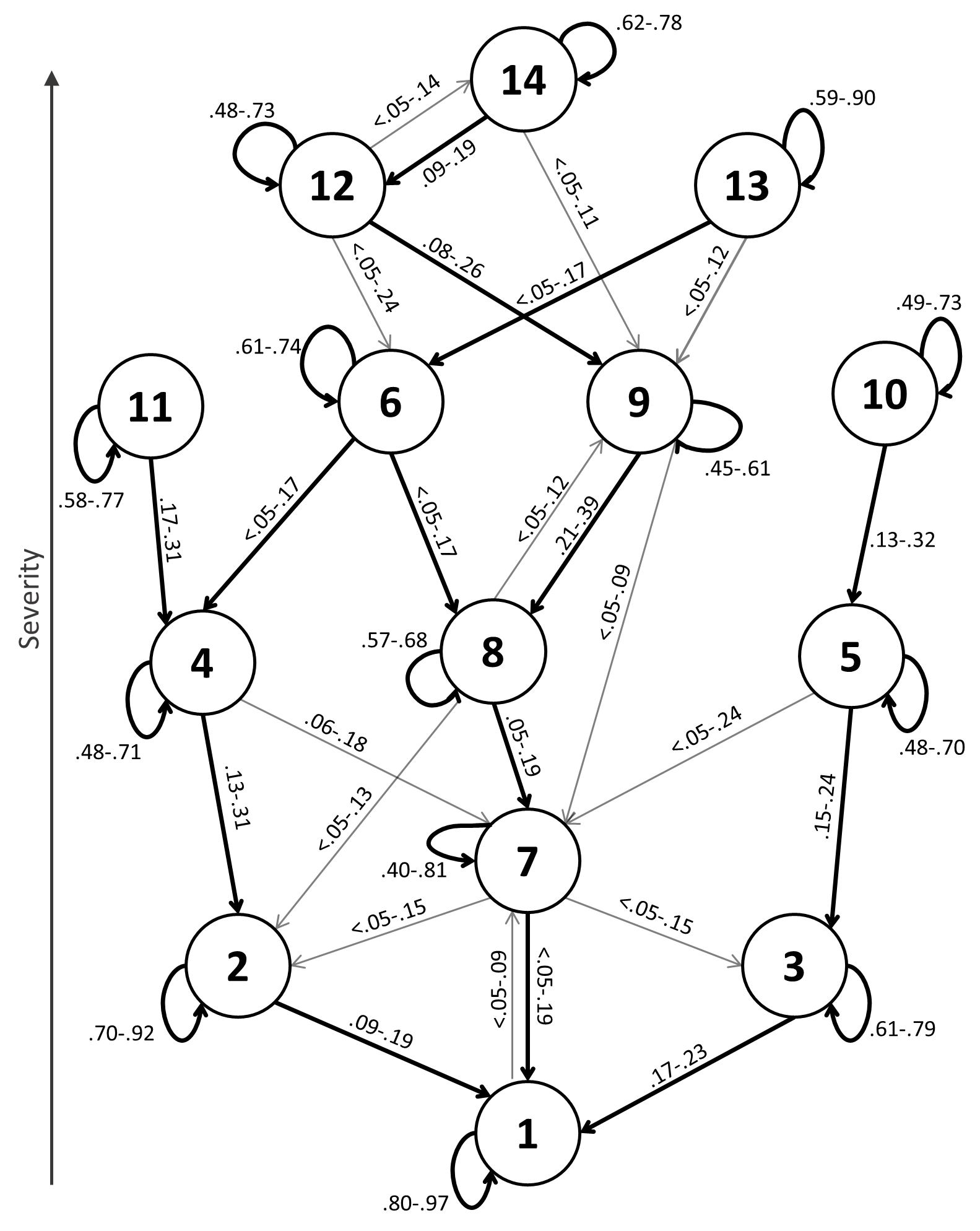


**Supplementary Figure 3 –** Transition probability graph showing range of transition probabilities across time for each depressive state, for the 14-state model; transition probabilities below .05 for more than half of the time points are omitted; thicker arrows represent the most likely transition between two given states. States 3, 5 and 10 represent cognitive/affective states (corresponding to single State 3 in the 7-state model); States 2, 4, 6 and 11 represent somatic states (corresponding to single State 5 in the 7-state model); State 6 represents a somatic depressive state with psychomotor retardation/agitation, while States 2, 4 and 11 represent somatic states without psychomotor retardation/agitation; States 7, 8 and 9 show relatively even severity across symptoms in the mild range (corresponding to State 4 in the 7-state model); States 12, 13 and 14 show even severity across symptoms in the moderate/severe range (corresponding to States 6 and 7 in the 7-state model); States 12 and 14 represent severe depressive states with suicidal ideation, while State 13 represents a severe depressive state without suicidal ideation.

| **Time** | **State** | **1** | **2** | **3** | **4** | **5** | **6** | **7** | **8** | **9** | **10** | **11** | **12** | **13** | **14** |
| --- | --- | --- | --- | --- | --- | --- | --- | --- | --- | --- | --- | --- | --- | --- | --- |
| **2** | **1** | 0.80 | <.005 | 0.09 | <.005 | <.005 | 0.02 | 0.09 | <.005 | <.005 | <.005 | <.005 | <.005 | <.005 | <.005 |
|  | **2** | 0.09 | 0.79 | 0.02 | 0.03 | <.005 | 0.03 | 0.02 | 0.02 | <.005 | <.005 | <.005 | <.005 | <.005 | <.005 |
|  | **3** | 0.19 | <.005 | 0.79 | <.005 | <.005 | <.005 | <.005 | 0.01 | <.005 | 0.01 | <.005 | <.005 | <.005 | <.005 |
|  | **4** | 0.01 | 0.13 | 0.01 | 0.68 | <.005 | 0.02 | 0.13 | <.005 | <.005 | <.005 | 0.03 | <.005 | <.005 | <.005 |
|  | **5** | 0.01 | 0.01 | 0.18 | <.005 | 0.63 | <.005 | 0.12 | 0.01 | 0.02 | 0.01 | <.005 | <.005 | <.005 | <.005 |
|  | **6** | <.005 | 0.04 | 0.01 | 0.06 | <.005 | 0.67 | <.005 | 0.16 | 0.03 | <.005 | <.005 | 0.01 | 0.02 | <.005 |
|  | **7** | 0.05 | 0.08 | 0.02 | <.005 | <.005 | <.005 | 0.81 | <.005 | <.005 | 0.03 | 0.01 | <.005 | <.005 | <.005 |
|  | **8** | 0.03 | 0.05 | 0.03 | <.005 | <.005 | 0.03 | 0.12 | 0.62 | 0.10 | <.005 | <.005 | <.005 | 0.01 | 0.01 |
|  | **9** | <.005 | <.005 | <.005 | <.005 | 0.05 | <.005 | 0.05 | 0.21 | 0.61 | <.005 | <.005 | 0.05 | 0.02 | 0.01 |
|  | **10** | <.005 | 0.01 | <.005 | <.005 | 0.32 | <.005 | <.005 | 0.01 | 0.02 | 0.60 | 0.01 | 0.02 | <.005 | <.005 |
|  | **11** | <.005 | 0.01 | <.005 | 0.29 | <.005 | <.005 | 0.03 | <.005 | 0.01 | <.005 | 0.65 | <.005 | <.005 | <.005 |
|  | **12** | <.005 | <.005 | <.005 | 0.01 | 0.01 | 0.06 | <.005 | 0.03 | 0.20 | <.005 | 0.02 | 0.64 | <.005 | 0.04 |
|  | **13** | <.005 | <.005 | <.005 | 0.01 | <.005 | 0.13 | 0.01 | 0.05 | 0.06 | <.005 | 0.06 | <.005 | 0.68 | 0.01 |
|  | **14** | <.005 | <.005 | <.005 | <.005 | 0.01 | 0.01 | <.005 | 0.02 | 0.02 | 0.01 | 0.04 | 0.18 | 0.02 | 0.69 |
|  |  |  |  |  |  |  |  |  |  |  |  |  |  |  |  |
| **Time** | **State** | **1** | **2** | **3** | **4** | **5** | **6** | **7** | **8** | **9** | **10** | **11** | **12** | **13** | **14** |
| **3** | **1** | 0.94 | <.005 | 0.05 | <.005 | 0.01 | <.005 | <.005 | <.005 | <.005 | <.005 | <.005 | <.005 | <.005 | <.005 |
|  | **2** | 0.14 | 0.82 | 0.01 | 0.02 | <.005 | <.005 | <.005 | 0.02 | <.005 | <.005 | <.005 | <.005 | <.005 | <.005 |
|  | **3** | 0.18 | 0.01 | 0.74 | <.005 | 0.06 | <.005 | <.005 | <.005 | <.005 | <.005 | <.005 | <.005 | <.005 | <.005 |
|  | **4** | <.005 | 0.18 | 0.01 | 0.58 | 0.01 | 0.02 | 0.18 | <.005 | <.005 | <.005 | 0.02 | <.005 | <.005 | <.005 |
|  | **5** | 0.03 | 0.01 | 0.21 | 0.02 | 0.57 | <.005 | 0.09 | 0.03 | 0.01 | 0.03 | 0.01 | <.005 | <.005 | <.005 |
|  | **6** | 0.01 | 0.05 | <.005 | 0.07 | <.005 | 0.62 | <.005 | 0.17 | 0.03 | <.005 | <.005 | 0.02 | 0.04 | <.005 |
|  | **7** | 0.08 | 0.04 | 0.09 | 0.04 | 0.02 | <.005 | 0.73 | <.005 | 0.01 | <.005 | <.005 | <.005 | <.005 | <.005 |
|  | **8** | 0.02 | 0.09 | 0.04 | <.005 | <.005 | 0.02 | 0.11 | 0.60 | 0.09 | <.005 | <.005 | 0.01 | 0.01 | <.005 |
|  | **9** | <.005 | 0.02 | 0.01 | <.005 | <.005 | 0.02 | 0.09 | 0.23 | 0.58 | <.005 | <.005 | 0.02 | 0.02 | 0.01 |
|  | **10** | 0.01 | <.005 | 0.04 | <.005 | 0.25 | 0.01 | <.005 | 0.02 | 0.04 | 0.57 | 0.02 | 0.03 | <.005 | <.005 |
|  | **11** | <.005 | 0.02 | <.005 | 0.28 | 0.01 | <.005 | 0.06 | <.005 | 0.01 | <.005 | 0.61 | <.005 | 0.01 | 0.01 |
|  | **12** | <.005 | <.005 | 0.01 | 0.01 | 0.01 | 0.06 | 0.01 | 0.05 | 0.18 | 0.01 | 0.02 | 0.61 | <.005 | 0.04 |
|  | **13** | 0.01 | 0.01 | <.005 | 0.01 | <.005 | 0.16 | 0.01 | 0.04 | 0.08 | <.005 | <.005 | <.005 | 0.67 | <.005 |
|  | **14** | <.005 | <.005 | <.005 | 0.01 | <.005 | 0.02 | 0.01 | 0.02 | 0.05 | 0.01 | 0.02 | 0.18 | 0.06 | 0.62 |
|  |  |  |  |  |  |  |  |  |  |  |  |  |  |  |  |
| **Time** | **State** | **1** | **2** | **3** | **4** | **5** | **6** | **7** | **8** | **9** | **10** | **11** | **12** | **13** | **14** |
| **4** | **1** | 0.86 | <.005 | 0.07 | <.005 | 0.02 | <.005 | 0.03 | <.005 | <.005 | <.005 | <.005 | 0.01 | <.005 | <.005 |
|  | **2** | 0.19 | 0.70 | 0.01 | 0.05 | <.005 | 0.01 | 0.01 | 0.02 | <.005 | <.005 | 0.01 | <.005 | <.005 | <.005 |
|  | **3** | 0.20 | 0.01 | 0.72 | <.005 | 0.06 | <.005 | <.005 | <.005 | 0.01 | <.005 | <.005 | <.005 | <.005 | <.005 |
|  | **4** | <.005 | 0.13 | <.005 | 0.67 | <.005 | <.005 | 0.13 | <.005 | 0.01 | <.005 | 0.05 | <.005 | <.005 | <.005 |
|  | **5** | <.005 | 0.03 | 0.22 | 0.01 | 0.59 | <.005 | 0.07 | 0.02 | <.005 | 0.06 | <.005 | <.005 | <.005 | <.005 |
|  | **6** | <.005 | 0.05 | <.005 | 0.09 | <.005 | 0.64 | 0.01 | 0.11 | 0.01 | <.005 | <.005 | <.005 | 0.07 | 0.02 |
|  | **7** | 0.06 | 0.11 | 0.12 | 0.04 | 0.04 | <.005 | 0.61 | 0.01 | 0.03 | <.005 | <.005 | <.005 | <.005 | <.005 |
|  | **8** | 0.01 | 0.06 | 0.03 | <.005 | 0.01 | 0.01 | 0.16 | 0.62 | 0.07 | 0.01 | <.005 | 0.01 | 0.01 | <.005 |
|  | **9** | 0.01 | 0.01 | <.005 | 0.02 | 0.04 | <.005 | 0.04 | 0.25 | 0.55 | <.005 | <.005 | 0.07 | <.005 | 0.01 |
|  | **10** | 0.02 | <.005 | 0.04 | <.005 | 0.30 | <.005 | 0.01 | 0.02 | 0.03 | 0.54 | 0.03 | <.005 | <.005 | 0.01 |
|  | **11** | <.005 | 0.02 | <.005 | 0.26 | <.005 | <.005 | 0.01 | <.005 | 0.01 | 0.01 | 0.65 | 0.01 | 0.02 | 0.01 |
|  | **12** | <.005 | 0.01 | <.005 | 0.01 | <.005 | 0.07 | 0.01 | 0.04 | 0.14 | <.005 | 0.02 | 0.60 | 0.03 | 0.07 |
|  | **13** | <.005 | 0.01 | 0.01 | 0.01 | 0.01 | 0.10 | 0.02 | 0.06 | 0.12 | 0.01 | 0.03 | <.005 | 0.59 | 0.03 |
|  | **14** | <.005 | <.005 | <.005 | <.005 | <.005 | <.005 | 0.01 | 0.03 | 0.05 | 0.03 | 0.04 | 0.13 | 0.02 | 0.68 |
|  |  |  |  |  |  |  |  |  |  |  |  |  |  |  |  |
| **Time** | **State** | **1** | **2** | **3** | **4** | **5** | **6** | **7** | **8** | **9** | **10** | **11** | **12** | **13** | **14** |
| **5** | **1** | 0.91 | <.005 | 0.05 | <.005 | <.005 | <.005 | 0.01 | 0.02 | <.005 | 0.01 | <.005 | <.005 | <.005 | <.005 |
|  | **2** | 0.09 | 0.81 | <.005 | 0.07 | 0.01 | <.005 | 0.01 | <.005 | <.005 | 0.01 | <.005 | <.005 | <.005 | <.005 |
|  | **3** | 0.20 | <.005 | 0.72 | <.005 | 0.04 | <.005 | 0.02 | 0.01 | <.005 | <.005 | <.005 | <.005 | <.005 | <.005 |
|  | **4** | <.005 | 0.17 | <.005 | 0.63 | <.005 | 0.02 | 0.11 | <.005 | <.005 | <.005 | 0.06 | <.005 | <.005 | 0.01 |
|  | **5** | 0.02 | <.005 | 0.23 | <.005 | 0.55 | <.005 | 0.12 | <.005 | 0.02 | 0.04 | 0.01 | <.005 | <.005 | <.005 |
|  | **6** | 0.01 | 0.06 | 0.01 | 0.07 | <.005 | 0.61 | <.005 | 0.13 | 0.05 | <.005 | 0.01 | 0.02 | 0.02 | <.005 |
|  | **7** | 0.06 | 0.12 | 0.13 | 0.03 | <.005 | <.005 | 0.61 | 0.01 | 0.02 | <.005 | <.005 | <.005 | <.005 | <.005 |
|  | **8** | 0.02 | 0.04 | 0.04 | 0.02 | 0.01 | 0.02 | 0.07 | 0.67 | 0.09 | <.005 | <.005 | <.005 | 0.01 | 0.01 |
|  | **9** | <.005 | 0.01 | <.005 | 0.01 | 0.01 | 0.02 | 0.08 | 0.28 | 0.50 | 0.02 | <.005 | 0.04 | 0.02 | 0.01 |
|  | **10** | 0.01 | <.005 | 0.03 | 0.01 | 0.20 | 0.01 | 0.04 | 0.02 | <.005 | 0.64 | <.005 | <.005 | <.005 | 0.03 |
|  | **11** | <.005 | 0.01 | 0.01 | 0.27 | 0.02 | 0.01 | <.005 | <.005 | <.005 | <.005 | 0.66 | 0.02 | 0.01 | <.005 |
|  | **12** | <.005 | <.005 | <.005 | 0.01 | <.005 | 0.05 | <.005 | 0.06 | 0.14 | <.005 | 0.01 | 0.66 | 0.01 | 0.05 |
|  | **13** | <.005 | 0.01 | <.005 | 0.05 | 0.01 | 0.11 | <.005 | 0.03 | 0.05 | <.005 | 0.02 | <.005 | 0.67 | 0.06 |
|  | **14** | <.005 | <.005 | <.005 | <.005 | <.005 | 0.05 | <.005 | 0.02 | <.005 | <.005 | 0.02 | 0.15 | 0.05 | 0.72 |
|  |  |  |  |  |  |  |  |  |  |  |  |  |  |  |  |
| **Time** | **State** | **1** | **2** | **3** | **4** | **5** | **6** | **7** | **8** | **9** | **10** | **11** | **12** | **13** | **14** |
| **6** | **1** | 0.86 | 0.02 | 0.09 | <.005 | <.005 | <.005 | 0.01 | <.005 | 0.01 | <.005 | 0.01 | <.005 | <.005 | <.005 |
|  | **2** | 0.13 | 0.75 | <.005 | 0.02 | <.005 | <.005 | 0.06 | 0.02 | 0.02 | <.005 | <.005 | <.005 | <.005 | <.005 |
|  | **3** | 0.19 | 0.02 | 0.72 | <.005 | 0.07 | <.005 | <.005 | <.005 | <.005 | <.005 | <.005 | 0.01 | <.005 | <.005 |
|  | **4** | <.005 | 0.20 | <.005 | 0.59 | 0.01 | 0.01 | 0.09 | 0.01 | 0.01 | <.005 | 0.08 | <.005 | <.005 | <.005 |
|  | **5** | <.005 | 0.02 | 0.21 | 0.01 | 0.70 | 0.01 | <.005 | <.005 | 0.01 | 0.04 | <.005 | <.005 | <.005 | <.005 |
|  | **6** | <.005 | 0.03 | <.005 | 0.10 | <.005 | 0.66 | <.005 | 0.15 | 0.01 | <.005 | 0.01 | 0.03 | 0.01 | <.005 |
|  | **7** | 0.06 | 0.09 | 0.15 | 0.02 | 0.01 | <.005 | 0.61 | 0.04 | 0.01 | <.005 | <.005 | <.005 | <.005 | <.005 |
|  | **8** | 0.02 | 0.10 | 0.05 | <.005 | <.005 | 0.02 | 0.11 | 0.59 | 0.09 | <.005 | <.005 | 0.01 | <.005 | 0.01 |
|  | **9** | 0.02 | <.005 | 0.02 | 0.01 | <.005 | <.005 | 0.06 | 0.28 | 0.53 | <.005 | 0.01 | 0.06 | <.005 | 0.01 |
|  | **10** | 0.06 | 0.02 | 0.04 | 0.07 | 0.20 | <.005 | 0.06 | <.005 | <.005 | 0.53 | <.005 | <.005 | <.005 | 0.03 |
|  | **11** | <.005 | <.005 | 0.01 | 0.17 | 0.04 | <.005 | <.005 | <.005 | <.005 | 0.01 | 0.77 | <.005 | <.005 | <.005 |
|  | **12** | <.005 | <.005 | 0.01 | 0.01 | 0.01 | <.005 | <.005 | 0.07 | 0.17 | <.005 | 0.01 | 0.57 | <.005 | 0.13 |
|  | **13** | <.005 | <.005 | 0.01 | <.005 | 0.01 | 0.06 | 0.01 | 0.04 | 0.12 | <.005 | 0.01 | <.005 | 0.73 | 0.01 |
|  | **14** | <.005 | 0.02 | <.005 | <.005 | <.005 | 0.05 | <.005 | 0.03 | 0.06 | <.005 | <.005 | 0.09 | <.005 | 0.75 |
|  |  |  |  |  |  |  |  |  |  |  |  |  |  |  |  |
| **Time** | **State** | **1** | **2** | **3** | **4** | **5** | **6** | **7** | **8** | **9** | **10** | **11** | **12** | **13** | **14** |
| **7** | **1** | 0.89 | 0.01 | 0.06 | <.005 | <.005 | <.005 | 0.01 | 0.02 | <.005 | <.005 | <.005 | <.005 | <.005 | <.005 |
|  | **2** | 0.16 | 0.71 | <.005 | 0.05 | 0.02 | 0.01 | <.005 | 0.04 | <.005 | <.005 | 0.01 | <.005 | <.005 | <.005 |
|  | **3** | 0.21 | <.005 | 0.72 | <.005 | 0.03 | <.005 | 0.02 | <.005 | 0.01 | <.005 | <.005 | <.005 | <.005 | <.005 |
|  | **4** | <.005 | 0.23 | <.005 | 0.71 | <.005 | <.005 | 0.06 | <.005 | 0.01 | <.005 | <.005 | <.005 | <.005 | <.005 |
|  | **5** | 0.02 | <.005 | 0.24 | <.005 | 0.67 | <.005 | <.005 | <.005 | 0.01 | 0.06 | <.005 | <.005 | <.005 | <.005 |
|  | **6** | 0.01 | 0.01 | <.005 | 0.02 | <.005 | 0.74 | 0.02 | 0.15 | <.005 | <.005 | <.005 | <.005 | 0.02 | 0.01 |
|  | **7** | 0.04 | 0.11 | 0.10 | 0.04 | 0.01 | <.005 | 0.66 | 0.02 | 0.01 | 0.03 | <.005 | <.005 | <.005 | <.005 |
|  | **8** | 0.02 | 0.09 | 0.07 | 0.01 | <.005 | 0.02 | 0.05 | 0.68 | 0.05 | <.005 | <.005 | <.005 | <.005 | 0.01 |
|  | **9** | 0.02 | <.005 | <.005 | <.005 | 0.01 | <.005 | 0.06 | 0.30 | 0.53 | <.005 | <.005 | 0.06 | 0.02 | <.005 |
|  | **10** | <.005 | <.005 | 0.05 | <.005 | 0.19 | <.005 | 0.03 | 0.01 | <.005 | 0.73 | <.005 | <.005 | <.005 | <.005 |
|  | **11** | <.005 | 0.02 | <.005 | 0.31 | 0.02 | <.005 | 0.02 | <.005 | <.005 | <.005 | 0.58 | <.005 | 0.03 | 0.02 |
|  | **12** | <.005 | <.005 | <.005 | <.005 | <.005 | 0.05 | <.005 | 0.05 | 0.15 | <.005 | <.005 | 0.70 | <.005 | 0.05 |
|  | **13** | <.005 | <.005 | <.005 | 0.01 | <.005 | 0.17 | 0.01 | 0.05 | 0.04 | <.005 | <.005 | <.005 | 0.72 | <.005 |
|  | **14** | <.005 | <.005 | 0.01 | <.005 | <.005 | 0.02 | 0.03 | 0.05 | 0.07 | <.005 | 0.01 | 0.11 | <.005 | 0.70 |
|  |  |  |  |  |  |  |  |  |  |  |  |  |  |  |  |
| **Time** | **State** | **1** | **2** | **3** | **4** | **5** | **6** | **7** | **8** | **9** | **10** | **11** | **12** | **13** | **14** |
| **8** | **1** | 0.89 | 0.01 | 0.08 | <.005 | <.005 | <.005 | 0.02 | <.005 | <.005 | <.005 | <.005 | <.005 | <.005 | <.005 |
|  | **2** | 0.14 | 0.78 | <.005 | <.005 | 0.01 | <.005 | 0.02 | 0.03 | <.005 | <.005 | 0.02 | <.005 | <.005 | <.005 |
|  | **3** | 0.23 | 0.01 | 0.72 | <.005 | 0.03 | 0.01 | <.005 | <.005 | <.005 | <.005 | <.005 | <.005 | <.005 | <.005 |
|  | **4** | 0.02 | 0.16 | 0.04 | 0.71 | <.005 | <.005 | <.005 | <.005 | <.005 | <.005 | 0.08 | <.005 | <.005 | <.005 |
|  | **5** | 0.01 | 0.02 | 0.15 | <.005 | 0.61 | <.005 | 0.16 | <.005 | 0.02 | 0.02 | <.005 | 0.01 | <.005 | <.005 |
|  | **6** | <.005 | 0.02 | <.005 | 0.14 | <.005 | 0.68 | <.005 | 0.07 | 0.01 | <.005 | 0.03 | 0.04 | 0.02 | <.005 |
|  | **7** | 0.02 | 0.12 | 0.10 | <.005 | <.005 | <.005 | 0.70 | <.005 | 0.03 | <.005 | 0.02 | <.005 | <.005 | 0.01 |
|  | **8** | <.005 | 0.11 | 0.05 | <.005 | <.005 | 0.01 | 0.11 | 0.66 | 0.04 | <.005 | <.005 | 0.01 | <.005 | 0.01 |
|  | **9** | <.005 | <.005 | 0.04 | 0.04 | <.005 | <.005 | 0.02 | 0.30 | 0.52 | <.005 | <.005 | 0.05 | <.005 | 0.02 |
|  | **10** | <.005 | <.005 | 0.04 | 0.05 | 0.17 | <.005 | 0.12 | <.005 | <.005 | 0.58 | <.005 | <.005 | <.005 | 0.04 |
|  | **11** | <.005 | <.005 | <.005 | 0.21 | 0.02 | <.005 | 0.10 | <.005 | <.005 | <.005 | 0.60 | 0.05 | 0.02 | <.005 |
|  | **12** | 0.01 | <.005 | <.005 | <.005 | 0.05 | <.005 | <.005 | 0.01 | 0.10 | <.005 | 0.02 | 0.73 | <.005 | 0.08 |
|  | **13** | <.005 | 0.05 | <.005 | <.005 | <.005 | 0.10 | 0.02 | <.005 | 0.09 | <.005 | <.005 | <.005 | 0.74 | <.005 |
|  | **14** | <.005 | 0.02 | <.005 | <.005 | 0.02 | <.005 | <.005 | 0.07 | 0.03 | <.005 | 0.06 | 0.17 | <.005 | 0.63 |
|  |  |  |  |  |  |  |  |  |  |  |  |  |  |  |  |
| **Time** | **State** | **1** | **2** | **3** | **4** | **5** | **6** | **7** | **8** | **9** | **10** | **11** | **12** | **13** | **14** |
| **9** | **1** | 0.88 | <.005 | 0.04 | <.005 | <.005 | <.005 | 0.06 | <.005 | 0.02 | <.005 | <.005 | <.005 | <.005 | <.005 |
|  | **2** | 0.10 | 0.83 | <.005 | 0.04 | 0.03 | <.005 | <.005 | <.005 | <.005 | <.005 | <.005 | <.005 | <.005 | <.005 |
|  | **3** | 0.22 | <.005 | 0.72 | <.005 | 0.04 | <.005 | <.005 | <.005 | <.005 | 0.01 | <.005 | <.005 | 0.01 | <.005 |
|  | **4** | 0.02 | 0.20 | 0.02 | 0.56 | <.005 | <.005 | 0.11 | <.005 | <.005 | <.005 | 0.09 | <.005 | <.005 | <.005 |
|  | **5** | <.005 | <.005 | 0.23 | <.005 | 0.69 | <.005 | 0.06 | <.005 | 0.01 | <.005 | <.005 | <.005 | <.005 | 0.02 |
|  | **6** | <.005 | 0.05 | <.005 | <.005 | <.005 | 0.74 | <.005 | 0.16 | <.005 | <.005 | <.005 | 0.05 | <.005 | <.005 |
|  | **7** | 0.08 | 0.08 | 0.10 | 0.04 | <.005 | <.005 | 0.65 | 0.05 | <.005 | <.005 | <.005 | <.005 | <.005 | <.005 |
|  | **8** | 0.02 | 0.13 | <.005 | 0.02 | <.005 | <.005 | 0.19 | 0.57 | 0.03 | <.005 | <.005 | 0.04 | 0.01 | <.005 |
|  | **9** | 0.04 | <.005 | 0.04 | <.005 | <.005 | 0.05 | <.005 | 0.22 | 0.60 | <.005 | <.005 | 0.06 | <.005 | <.005 |
|  | **10** | <.005 | <.005 | 0.04 | <.005 | 0.13 | <.005 | <.005 | <.005 | 0.31 | 0.49 | 0.03 | <.005 | <.005 | <.005 |
|  | **11** | <.005 | 0.04 | <.005 | 0.17 | <.005 | <.005 | 0.17 | <.005 | 0.02 | <.005 | 0.58 | 0.03 | <.005 | <.005 |
|  | **12** | <.005 | <.005 | <.005 | 0.02 | <.005 | 0.24 | <.005 | 0.03 | 0.08 | 0.01 | <.005 | 0.48 | <.005 | 0.14 |
|  | **13** | <.005 | <.005 | <.005 | <.005 | <.005 | <.005 | <.005 | 0.05 | <.005 | <.005 | <.005 | <.005 | 0.90 | 0.05 |
|  | **14** | <.005 | 0.03 | <.005 | <.005 | 0.04 | <.005 | <.005 | <.005 | <.005 | 0.02 | <.005 | 0.09 | 0.04 | 0.78 |
|  |  |  |  |  |  |  |  |  |  |  |  |  |  |  |  |
| **Time** | **State** | **1** | **2** | **3** | **4** | **5** | **6** | **7** | **8** | **9** | **10** | **11** | **12** | **13** | **14** |
| **10** | **1** | 0.97 | <.005 | <.005 | <.005 | <.005 | <.005 | 0.03 | <.005 | <.005 | <.005 | <.005 | <.005 | <.005 | <.005 |
|  | **2** | 0.04 | 0.92 | <.005 | 0.03 | <.005 | <.005 | <.005 | <.005 | <.005 | <.005 | <.005 | <.005 | <.005 | <.005 |
|  | **3** | 0.17 | 0.14 | 0.61 | <.005 | <.005 | <.005 | 0.05 | <.005 | <.005 | <.005 | <.005 | <.005 | <.005 | 0.02 |
|  | **4** | <.005 | 0.31 | 0.01 | 0.48 | <.005 | <.005 | 0.18 | <.005 | <.005 | <.005 | 0.02 | <.005 | <.005 | <.005 |
|  | **5** | 0.02 | <.005 | 0.23 | <.005 | 0.48 | <.005 | 0.24 | <.005 | <.005 | 0.03 | <.005 | <.005 | <.005 | <.005 |
|  | **6** | <.005 | <.005 | <.005 | 0.17 | <.005 | 0.71 | <.005 | 0.04 | <.005 | 0.01 | <.005 | <.005 | 0.04 | 0.03 |
|  | **7** | 0.19 | 0.15 | 0.15 | <.005 | 0.08 | <.005 | 0.40 | <.005 | <.005 | <.005 | 0.02 | <.005 | <.005 | <.005 |
|  | **8** | <.005 | 0.04 | <.005 | <.005 | 0.02 | 0.03 | 0.13 | 0.64 | 0.12 | <.005 | <.005 | <.005 | 0.02 | 0.02 |
|  | **9** | <.005 | 0.05 | <.005 | 0.06 | <.005 | 0.04 | <.005 | 0.39 | 0.45 | <.005 | <.005 | <.005 | <.005 | <.005 |
|  | **10** | 0.14 | <.005 | <.005 | <.005 | 0.28 | <.005 | <.005 | <.005 | <.005 | 0.58 | <.005 | <.005 | <.005 | <.005 |
|  | **11** | <.005 | <.005 | 0.08 | 0.25 | <.005 | <.005 | <.005 | <.005 | <.005 | 0.01 | 0.65 | <.005 | <.005 | <.005 |
|  | **12** | <.005 | <.005 | <.005 | <.005 | <.005 | 0.08 | <.005 | 0.03 | 0.26 | <.005 | <.005 | 0.51 | <.005 | 0.13 |
|  | **13** | 0.13 | <.005 | <.005 | <.005 | <.005 | 0.09 | 0.07 | <.005 | 0.11 | <.005 | 0.05 | <.005 | 0.54 | <.005 |
|  | **14** | <.005 | <.005 | <.005 | <.005 | <.005 | <.005 | <.005 | <.005 | 0.11 | <.005 | <.005 | 0.19 | <.005 | 0.70 |

**Supplementary Table 2 –** Transition probability matrix showing transition probabilities between starting state (rows) and end state (columns) at each time point, for the 14-state model

| **Time** | **State** | **1** | **2** | **3** | **4** | **5** | **6** | **7** |
| --- | --- | --- | --- | --- | --- | --- | --- | --- |
| **2** | **1** | 0.90 | 0.08 | <.005 | <.005 | 0.01 | 0.01 | <.005 |
|  | **2** | 0.16 | 0.79 | 0.03 | 0.02 | <.005 | <.005 | <.005 |
|  | **3** | 0.01 | 0.19 | 0.73 | 0.05 | <.005 | 0.01 | <.005 |
|  | **4** | 0.01 | 0.23 | <.005 | 0.68 | <.005 | 0.06 | 0.01 |
|  | **5** | 0.03 | 0.16 | <.005 | 0.02 | 0.78 | 0.01 | <.005 |
|  | **6** | <.005 | <.005 | 0.01 | 0.25 | 0.07 | 0.63 | 0.04 |
|  | **7** | <.005 | 0.01 | 0.03 | 0.03 | 0.03 | 0.23 | 0.66 |
|  |  |  |  |  |  |  |  |  |
| **Time** | **State** | 1 | 2 | 3 | 4 | 5 | 6 | 7 |
| **3** | **1** | 0.93 | 0.06 | <.005 | <.005 | 0.01 | <.005 | <.005 |
|  | **2** | 0.21 | 0.73 | 0.03 | 0.02 | 0.01 | <.005 | <.005 |
|  | **3** | 0.03 | 0.23 | 0.65 | 0.05 | 0.01 | 0.01 | 0.01 |
|  | **4** | 0.03 | 0.25 | <.005 | 0.63 | 0.01 | 0.07 | 0.01 |
|  | **5** | 0.03 | 0.20 | <.005 | 0.04 | 0.73 | <.005 | 0.01 |
|  | **6** | 0.01 | 0.03 | <.005 | 0.24 | 0.07 | 0.59 | 0.06 |
|  | **7** | 0.01 | 0.01 | 0.02 | 0.05 | 0.03 | 0.24 | 0.64 |
|  |  |  |  |  |  |  |  |  |
| **Time** | **State** | 1 | 2 | 3 | 4 | 5 | 6 | 7 |
| **4** | **1** | 0.91 | 0.04 | 0.02 | 0.01 | 0.02 | <.005 | 0.01 |
|  | **2** | 0.21 | 0.71 | 0.04 | 0.03 | 0.01 | <.005 | <.005 |
|  | **3** | 0.02 | 0.20 | 0.70 | 0.03 | 0.03 | 0.01 | 0.02 |
|  | **4** | 0.01 | 0.24 | <.005 | 0.66 | <.005 | 0.08 | 0.01 |
|  | **5** | 0.05 | 0.15 | <.005 | 0.02 | 0.73 | 0.03 | 0.02 |
|  | **6** | 0.01 | 0.03 | 0.01 | 0.23 | 0.05 | 0.59 | 0.08 |
|  | **7** | <.005 | 0.02 | 0.03 | 0.06 | 0.03 | 0.19 | 0.67 |
|  |  |  |  |  |  |  |  |  |
| **Time** | **State** | 1 | 2 | 3 | 4 | 5 | 6 | 7 |
| **5** | **1** | 0.92 | 0.07 | <.005 | 0.01 | <.005 | <.005 | <.005 |
|  | **2** | 0.19 | 0.72 | 0.04 | 0.03 | 0.01 | <.005 | <.005 |
|  | **3** | 0.03 | 0.27 | 0.64 | 0.01 | 0.02 | 0.01 | 0.01 |
|  | **4** | 0.01 | 0.21 | <.005 | 0.69 | 0.01 | 0.06 | 0.01 |
|  | **5** | 0.05 | 0.15 | <.005 | 0.02 | 0.74 | 0.02 | 0.02 |
|  | **6** | <.005 | 0.04 | <.005 | 0.22 | 0.06 | 0.60 | 0.08 |
|  | **7** | <.005 | <.005 | 0.02 | 0.03 | 0.05 | 0.16 | 0.74 |
|  |  |  |  |  |  |  |  |  |
| **Time** | **State** | 1 | 2 | 3 | 4 | 5 | 6 | 7 |
| **6** | **1** | 0.91 | 0.08 | 0.01 | <.005 | <.005 | <.005 | <.005 |
|  | **2** | 0.22 | 0.69 | 0.02 | 0.04 | 0.01 | 0.01 | <.005 |
|  | **3** | 0.04 | 0.23 | 0.66 | <.005 | 0.05 | 0.01 | 0.02 |
|  | **4** | 0.03 | 0.24 | <.005 | 0.62 | 0.01 | 0.10 | 0.01 |
|  | **5** | 0.05 | 0.13 | <.005 | 0.03 | 0.75 | 0.03 | 0.01 |
|  | **6** | <.005 | 0.04 | <.005 | 0.25 | 0.02 | 0.61 | 0.08 |
|  | **7** | <.005 | 0.01 | 0.01 | 0.06 | 0.01 | 0.20 | 0.71 |
|  |  |  |  |  |  |  |  |  |
| **Time** | **State** | 1 | 2 | 3 | 4 | 5 | 6 | 7 |
| **7** | **1** | 0.93 | 0.05 | <.005 | 0.02 | 0.01 | <.005 | <.005 |
|  | **2** | 0.22 | 0.68 | 0.05 | 0.04 | 0.01 | 0.01 | <.005 |
|  | **3** | 0.03 | 0.24 | 0.69 | <.005 | <.005 | <.005 | 0.03 |
|  | **4** | 0.04 | 0.20 | 0.01 | 0.68 | 0.02 | 0.04 | 0.01 |
|  | **5** | 0.02 | 0.17 | <.005 | 0.01 | 0.79 | <.005 | 0.01 |
|  | **6** | <.005 | 0.02 | <.005 | 0.26 | 0.05 | 0.63 | 0.04 |
|  | **7** | <.005 | 0.01 | 0.01 | 0.06 | 0.01 | 0.19 | 0.71 |
|  |  |  |  |  |  |  |  |  |
| **Time** | **State** | 1 | 2 | 3 | 4 | 5 | 6 | 7 |
| **8** | **1** | 0.91 | 0.07 | 0.01 | <.005 | <.005 | <.005 | <.005 |
|  | **2** | 0.20 | 0.74 | 0.01 | 0.02 | 0.01 | 0.02 | <.005 |
|  | **3** | 0.03 | 0.31 | 0.57 | 0.05 | 0.01 | <.005 | 0.03 |
|  | **4** | 0.02 | 0.21 | <.005 | 0.69 | <.005 | 0.05 | 0.02 |
|  | **5** | 0.05 | 0.13 | <.005 | <.005 | 0.78 | 0.02 | 0.01 |
|  | **6** | <.005 | 0.04 | <.005 | 0.19 | 0.04 | 0.64 | 0.08 |
|  | **7** | 0.01 | 0.01 | 0.01 | 0.08 | 0.02 | 0.19 | 0.68 |
|  |  |  |  |  |  |  |  |  |
| **Time** | **State** | 1 | 2 | 3 | 4 | 5 | 6 | 7 |
| **9** | **1** | 0.90 | 0.07 | <.005 | 0.01 | <.005 | 0.01 | <.005 |
|  | **2** | 0.23 | 0.74 | 0.02 | 0.02 | <.005 | <.005 | <.005 |
|  | **3** | <.005 | 0.32 | 0.64 | 0.01 | <.005 | 0.01 | 0.02 |
|  | **4** | 0.01 | 0.27 | <.005 | 0.64 | <.005 | 0.06 | 0.02 |
|  | **5** | 0.06 | 0.18 | <.005 | <.005 | 0.74 | 0.02 | <.005 |
|  | **6** | 0.02 | 0.02 | <.005 | 0.19 | 0.08 | 0.58 | 0.11 |
|  | **7** | 0.02 | <.005 | 0.04 | 0.02 | 0.05 | 0.07 | 0.80 |
|  |  |  |  |  |  |  |  |  |
| **Time** | **State** | 1 | 2 | 3 | 4 | 5 | 6 | 7 |
| **10** | **1** | 0.94 | 0.05 | <.005 | <.005 | 0.01 | <.005 | <.005 |
|  | **2** | 0.29 | 0.68 | 0.02 | <.005 | <.005 | <.005 | 0.01 |
|  | **3** | 0.07 | 0.32 | 0.49 | 0.12 | <.005 | <.005 | <.005 |
|  | **4** | 0.03 | 0.25 | 0.02 | 0.61 | <.005 | 0.07 | 0.01 |
|  | **5** | 0.03 | 0.14 | <.005 | 0.01 | 0.73 | 0.01 | 0.09 |
|  | **6** | 0.03 | <.005 | <.005 | 0.24 | 0.15 | 0.57 | 0.02 |
|  | **7** | 0.04 | 0.04 | <.005 | <.005 | <.005 | 0.45 | 0.47 |

**Supplementary Table 3 –** Transition probability matrix showing transition probabilities between starting state (rows) and end state (columns) at each time point, for the 7 state model.


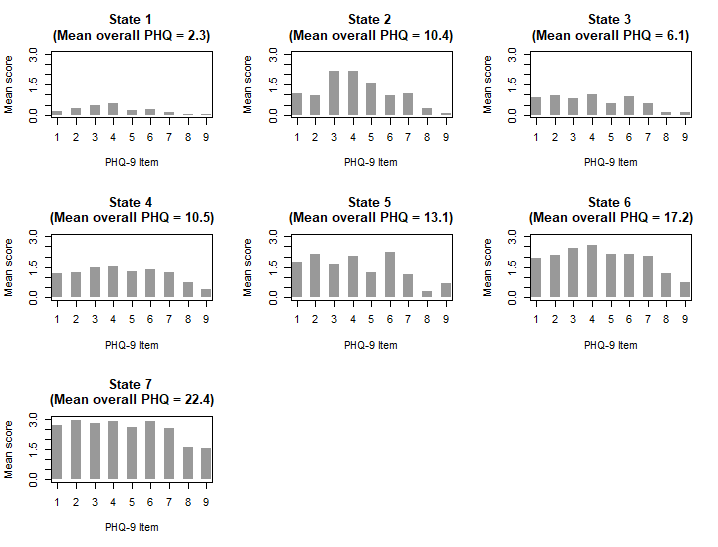

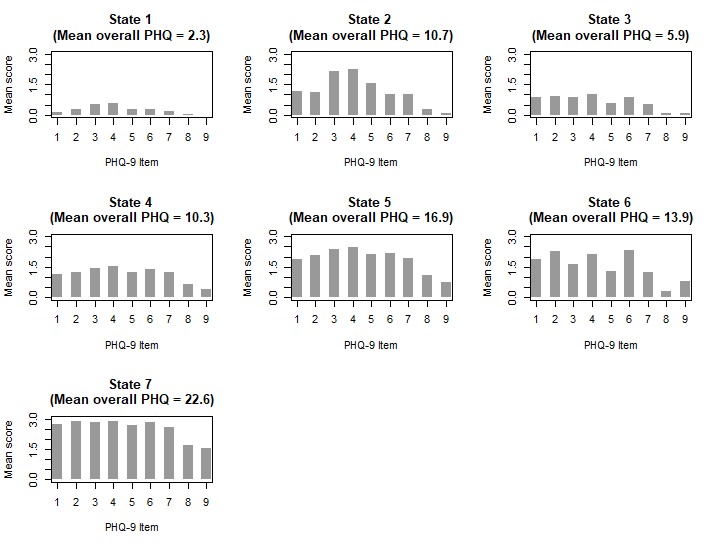


Sample 2

N = 3234

Sample 1

N = 3285


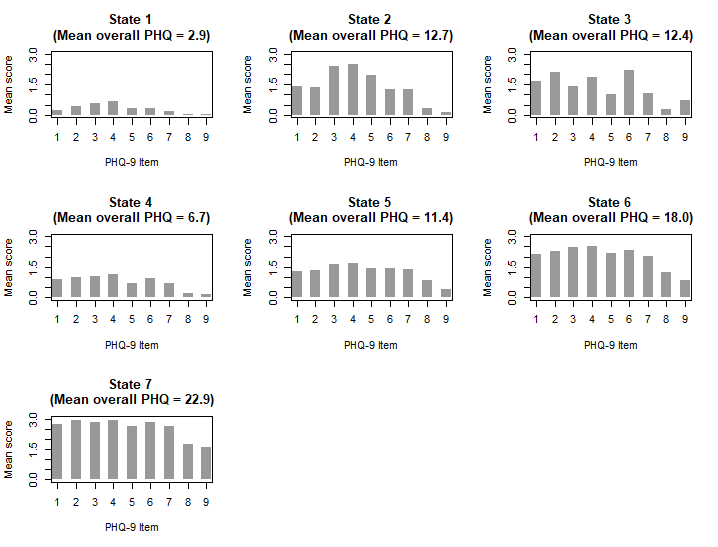


Sample 3

N = 3372

**Supplementary Figure 4 -** Graphical summary of state symptom profiles for the 7-state model, ran on three independent random samples of data (Sample 1: N = 3285; Sample 2: N = 3234; Sample 3: N = 3372). States 1 and 3 in samples 1 and 2, and States 1 and 4 in sample 3 represent states of minimal to mild overall severity; States 6, 5 and 3 in samples 1, 2 and 3 respectively shows peak symptom intensity around feelings of depression, tiredness and low self-esteem (cognitive/affective state); State 2 in all samples shows peak symptom intensity around difficulties sleeping, feelings of tiredness and changes in appetite (somatic state); State 4 in samples 1 and 2 and State 5 in sample 3 show a relatively even spread in symptom intensity across items (hybrid state); States 5 and 6 in sample 1 and States 6 and 7 in samples 2 and 3 represent moderately severe and severe states.


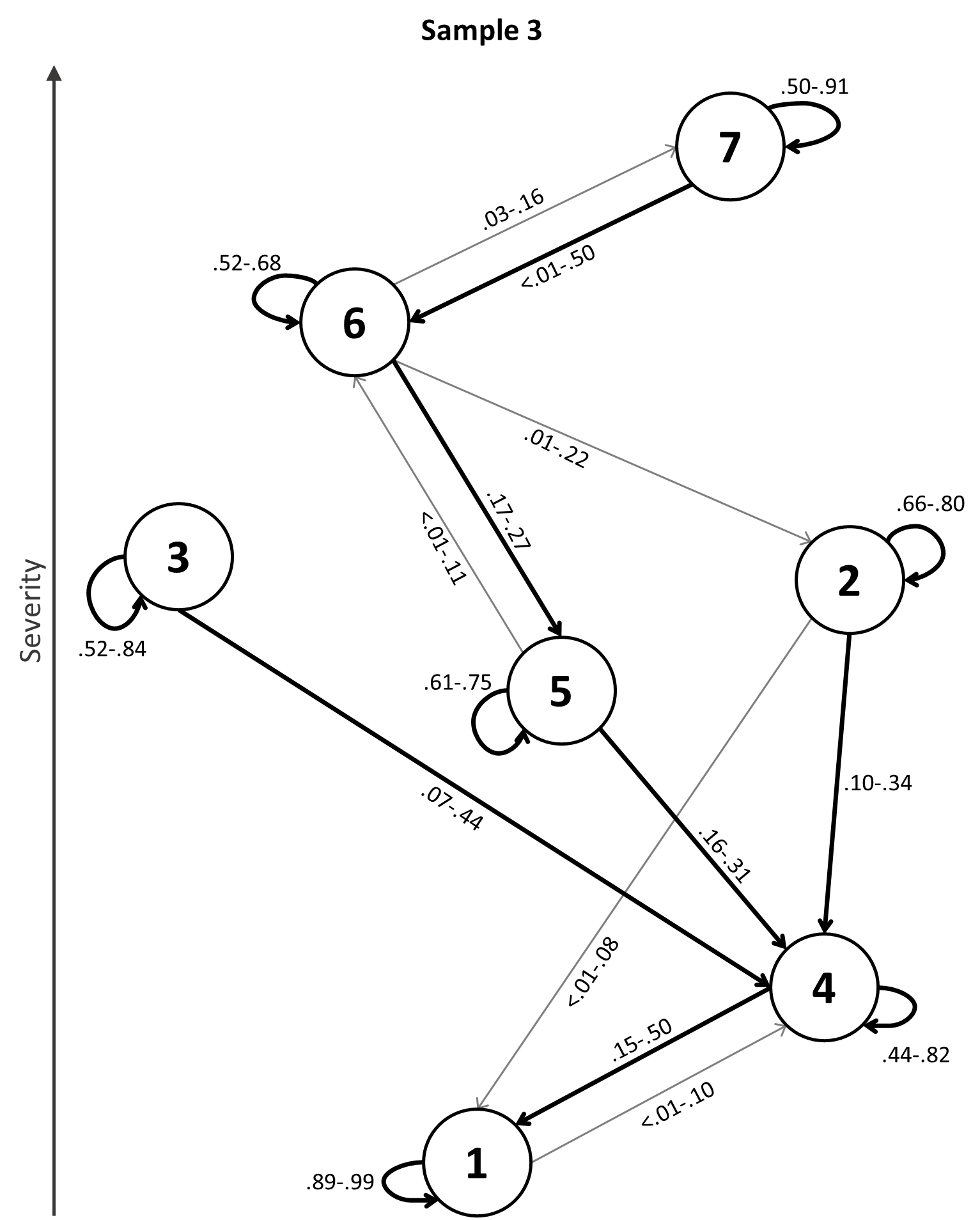

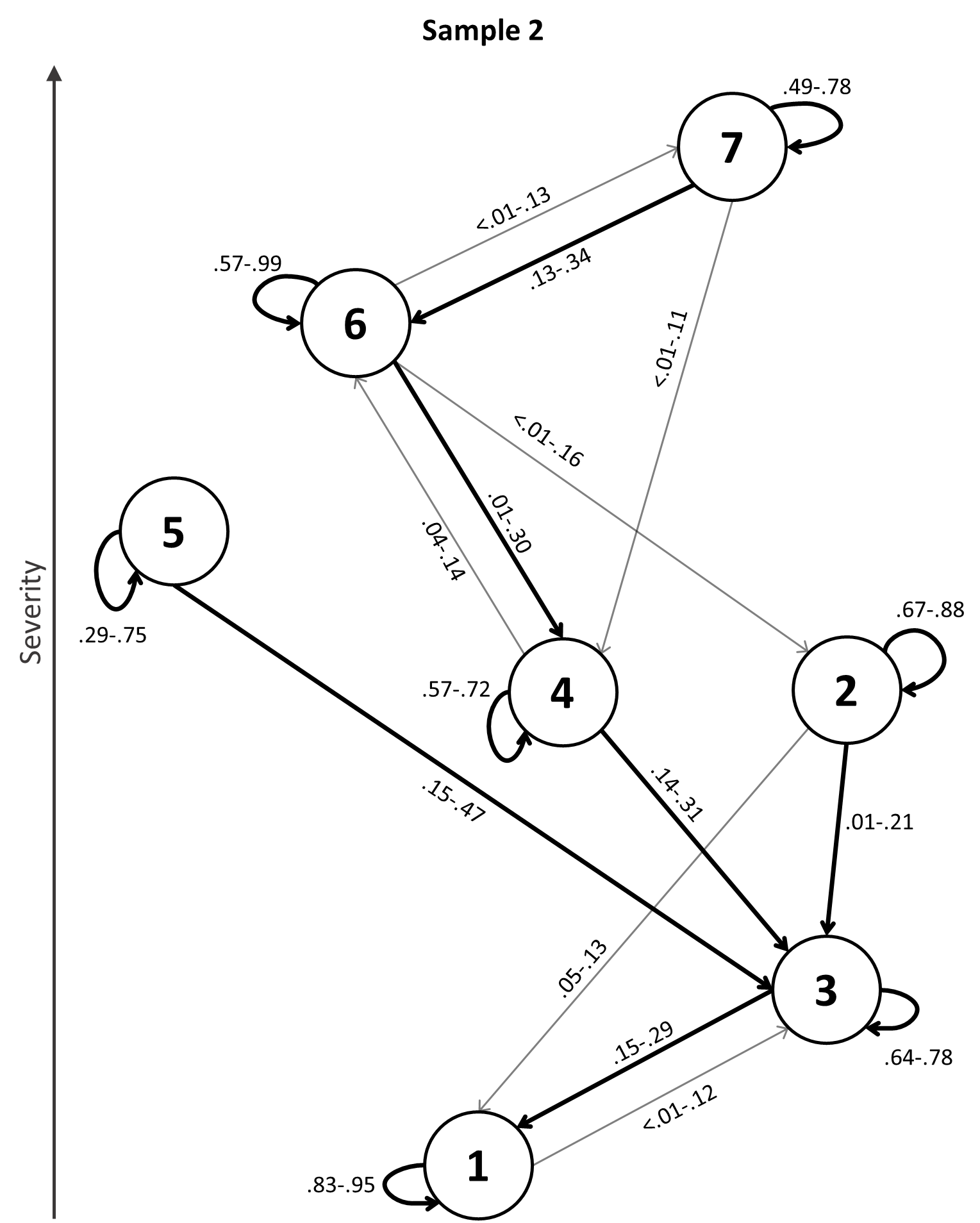

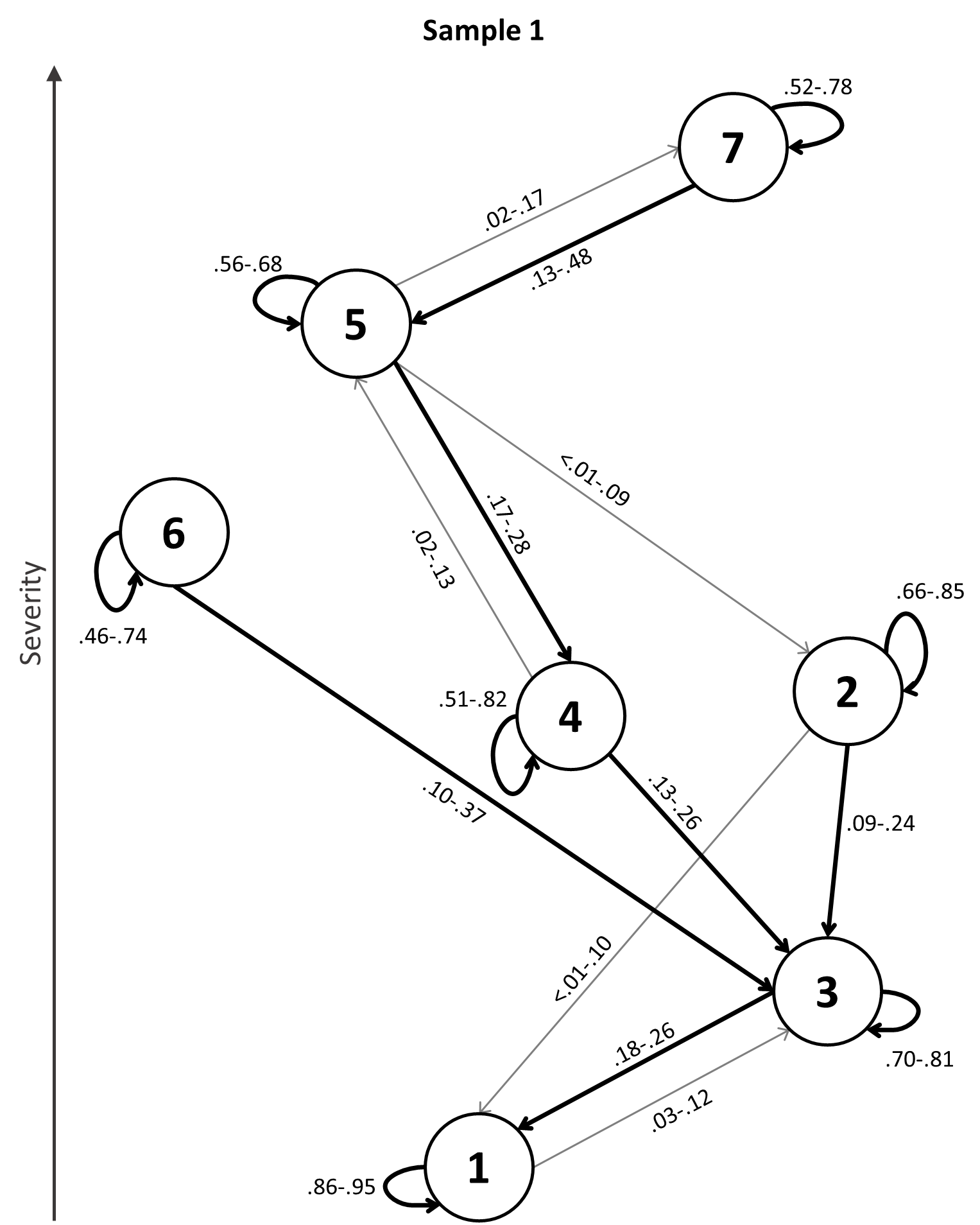


**Supplementary Figure 5 –** Transition probability graphs showing range of transition probabilities across time for each depressive state, for Samples 1, 2 and 3; transition probabilities below .05 for more than half of the time points are omitted; thicker arrows represent the most likely transitions between two given states. States 1 and 3 in samples 1 and 2, and States 1 and 4 in sample 3 represent states of minimal to mild overall severity; States 6, 5 and 3 in samples 1, 2 and 3 respectively shows peak symptom intensity around feelings of depression, tiredness and low self-esteem (cognitive/affective state); State 2 in all samples shows peak symptom intensity around difficulties sleeping, feelings of tiredness and changes in appetite (somatic state); State 4 in samples 1 and 2 and State 5 in sample 3 show a relatively even spread in symptom intensity across items (hybrid state); States 5 and 6 in sample 1 and States 6 and 7 in samples 2 and 3 represent moderately severe and severe states.
